# Supplementary material for: Atomically dispersed palladium catalyses Suzuki–Miyaura reactions under phosphine-free conditions
Source: Commun Chem. 2020 Apr 3;3:43. doi: 10.1038/s42004-020-0289-y (PMC9814916; doi:10.1038/s42004-020-0289-y)
Supplement: Supplementary file 2 — Supplementary Information [file 42004_2020_289_MOESM2_ESM.pdf]

Supporting Information For

Atomically-dispersed palladium catalyses Suzuki-Miyaura reactions under phosphine-free conditions

1. Optimize reaction conditions.

Supplementary Table 1 Screening of reaction conditions.[a]

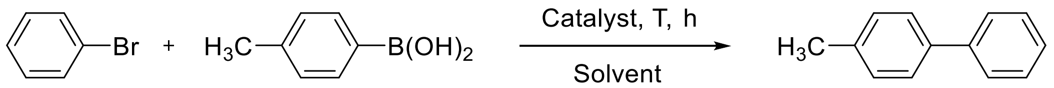

Entry	Solvents	Bases	Pd/mg(ppm)	Time/h	Yield/%[b]
1	EtOH	K_2CO_3	34(63)	20	15
2	MeOH	K_2CO_3	34(63)	20	34
3	H_2O	K_2CO_3	34(63)	20	78[c]
4	EtOH/H_2O	K_2CO_3	34(63)	3.5	98
5	MeOH/H_2O	K_2CO_3	34(63)	3.5	98
6	EtOH/H_2O	-	34(63)	3.5	<0.1
7	EtOH/H_2O	K_2CO_3	20(37)	4	99
8	EtOH/H_2O	K_2CO_3	2(3.8)	14	95
9	EtOH/H_2O	K_2CO_3	1.1(2)	14	43
10	EtOH/H_2O	K_2CO_3	-	14	0
11	EtOH/H_2O	K_2CO_3	-[d]	14	0

[a] All reactions were performed using 0.25 mmol of bromobenzene, 0.31 mmol of 4-methyl phenylboronic acid, 0.75 mmol of K_2CO_3, Pd-ZnO-ZrO_2, 5.0 mL of solvent (3/2 volume ratio if mixture solvents), 25 ^{o}C, under air conditions [b] GC yield based on the bromobenzene, using n-octane as an internal standard. [c] 12.2% 4,4'-dimethylbiphenyl was detected. [d] ZnO-ZrO_2 As catalyst.

Supplementary Table 2 Screening of reaction solvent.[a]

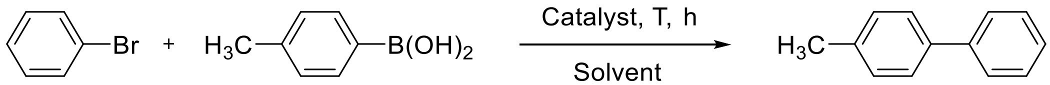

Entry	Solvent	Time/h	Yield/%[b]
1	DMF	5	0
2	Dioxane	5	0
3	Toluene	5	0
4	Et_2O	5	0
5	H_2O	5/20	25.9/77.8[c]
6	EtOH	5/20	3.8/14.8
7	MeOH	5/20	14.0/34.4
8	2-Propanol	5	0
9	DMF/H_2O	3.5	0.9
10	Dioxane/H_2O	3.5	56
11	Toluene/H_2O	3.5	0
12	Et_2O/H_2O	3.5	0.7
13	EtOH/H_2O	3.5	98
14	MeOH/H_2O	3.5	98

[a] All reactions were performed using 0.25 mmol of bromobenzene, 0.31 mmol of 4-methyl phenylboronic acid, 0.75 mmol of K_2CO_3, 20 mg (37 ppm) Pd catalyst, 5.0 mL of solvent, 25 ^oC. [b] GC yield based on the bromobenzene, using n-octane as an internal standard. [c] 12.2% 4,4'-Dimethylbiphenyl was detected.

Supplementary Table 3 Screening of different bases.[a]

Entry	Base	Yield/%[b]
1	K_2CO_3	98
2	Et_3N	22
3	Na_2CO_3	92
4	$NaHCO_3$	46
5	NaOH	23
6	KOH	33
7	-	< 0.1

[a] All reactions were performed using 0.25 mmol of bromobenzene, 0.31 mmol of 4-methyl phenylboronic acid, 0.75 mmol of the base, 20 mg (37 ppm) Pd catalyst, 5.0 mL of solvent(EtOH/H_2O: 3/2), 25 ^{o}C, 3.5 hours, under air condition. [b] GC yield based on the bromobenzene, using n-octane as an internal standard.

Supplementary Table 4 Catalyst loading effect[a]

Entry	Catalyst/mg/ppm Pd	Time/h	Conversion/%[b]	Yield/%[c]	TON[d]
1	34 mg (63ppm)	3.5	98.6	98.1	3913
2	20 mg (37ppm)	4	100	99.2	6758
3	10 mg (18ppm)	2	73.7	73.6	10236
4	5 mg (9.4 ppm)	2	56.0	55.6	14894
5	2 mg (3.8ppm)	14	95.1	94.6	62565
6	1.1 mg (2.1ppm)	14	43.3	42.9	51548

[a] All reactions were performed using 0.25 mmol of bromobenzene, 0.31 mmol of 4-methyl phenylboronic acid, 0.75 mmol of K_2CO_3, 5.0 mL of solvent(EtOH/H_2O: 3/2), 25 ^oC, under air conditions.[b] Reaction conversion was calculated through GC based on bromobenzene [c] Reaction yield was calculated through GC based on the bromobenzene using n-Octane as an internal standard. [d] TON was calculated using equation: $TON = \frac{mole\ of\ converted\ bromobenzne}{\text{mole of Pd catalyst}} \times 100\%$.

2. Characterization

Supplementary Table 5 The fit result of EXAFS data. Pd-O shell structure derived from EXAFS measurements. CN: coordination number; R: bond distance in R-space; σ^2: Debye-Waller factor; E_0: threshold energy.

Edge	Sample name	Paths	R (Å)	CN	Debye Waller factor(Å2)	Energy shift ΔE (eV)	R-factor
**Pd k-edge**<br>**So2=0.9**	Pd-ZnO-ZrO_2	Pd-O	2.017 ± 0.004	2.15 ± 0.27	0.0021	3.54 ± 1.74	0.000606

1 Note that no Pd-Pd shell was detected in Pd-ZnO-ZrO_2, indicating no Pd NPs in the sample.

2 Note that the Pd-O path was used to fit the first shell.

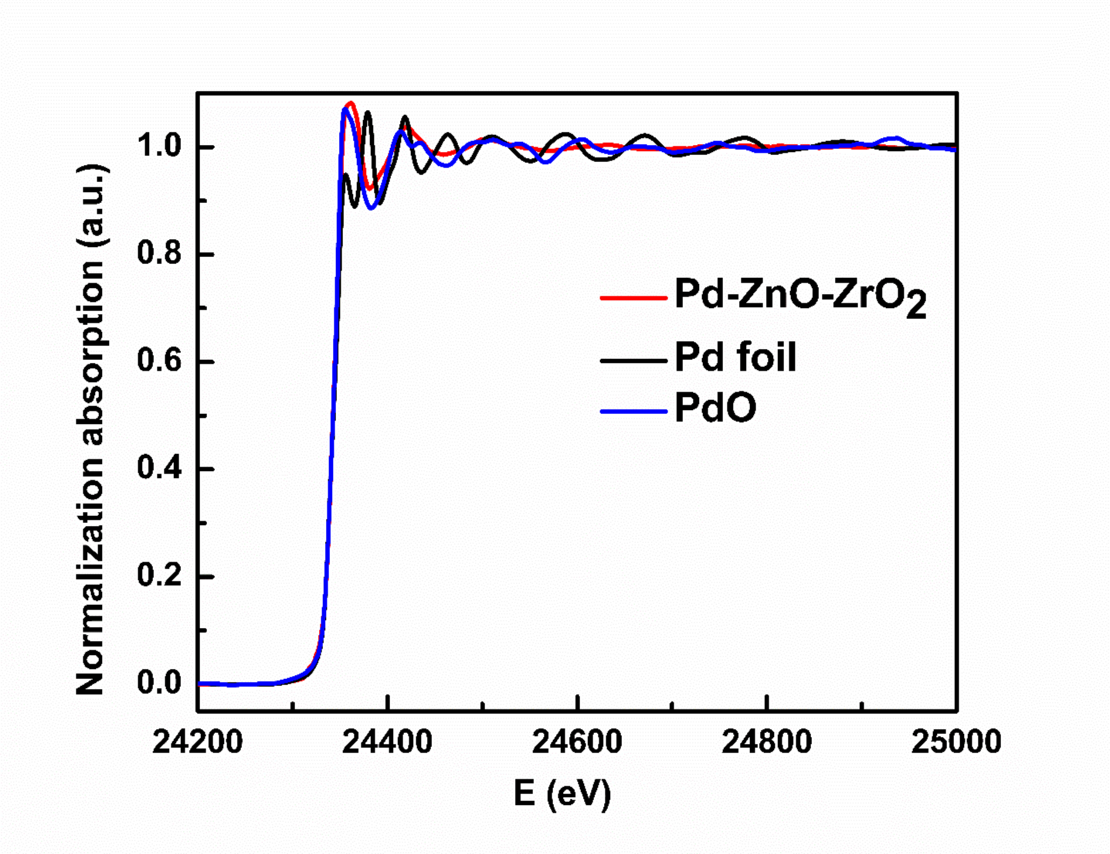

Supplementary Figure 1 XANES full spectra of the samples.

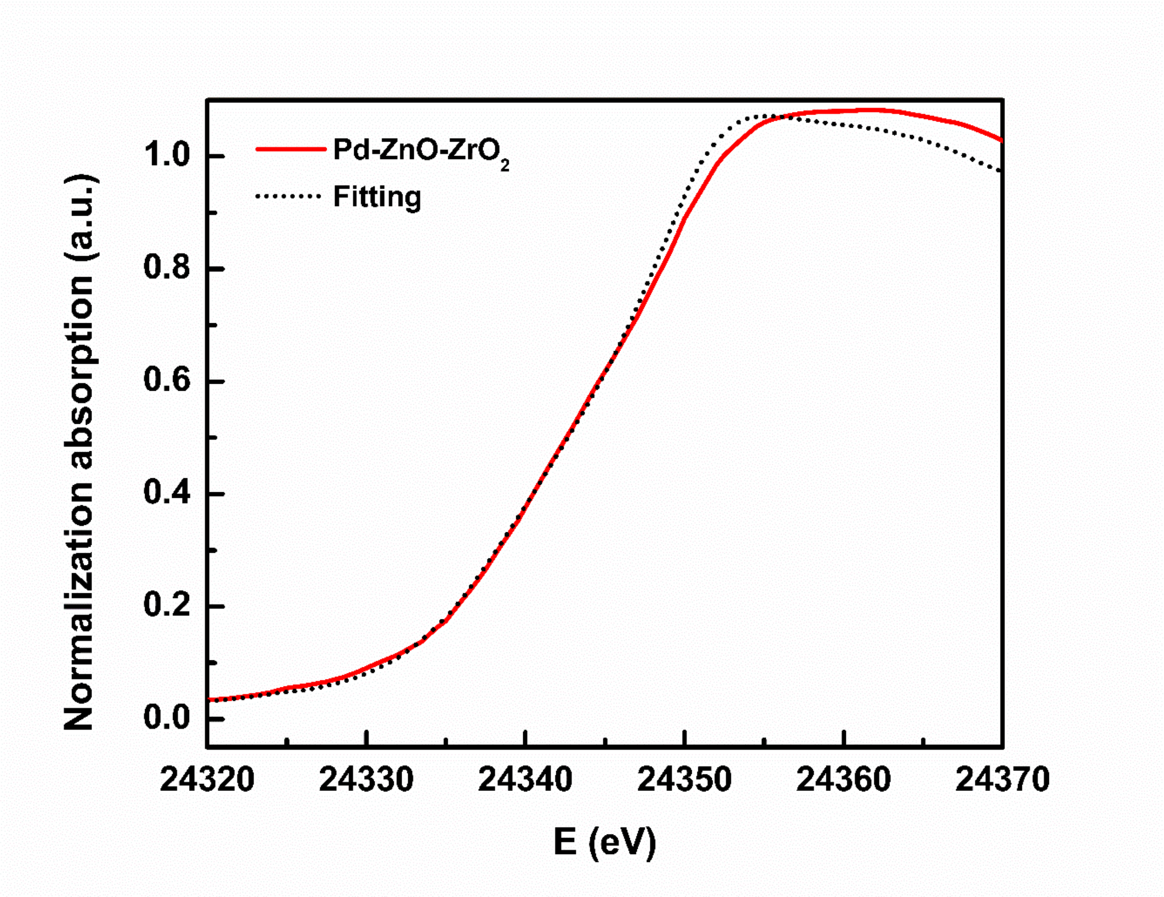

Supplementary Figure 2 Linear combination fitting of the samples.

Supplementary Table 6 The linear combination fitting method to fit the chemical status of Pd-ZnO-ZrO_2 samples with PdO and Pd foil as references.

Pd-ZnO-ZrO_2	PdO (Ref)	Pd Foil (Ref)	R-factor	Chi-square
	100%	0%	0.00556	0.00089

Pd foil did not make any contribution to the chemical status based on the fitting results.

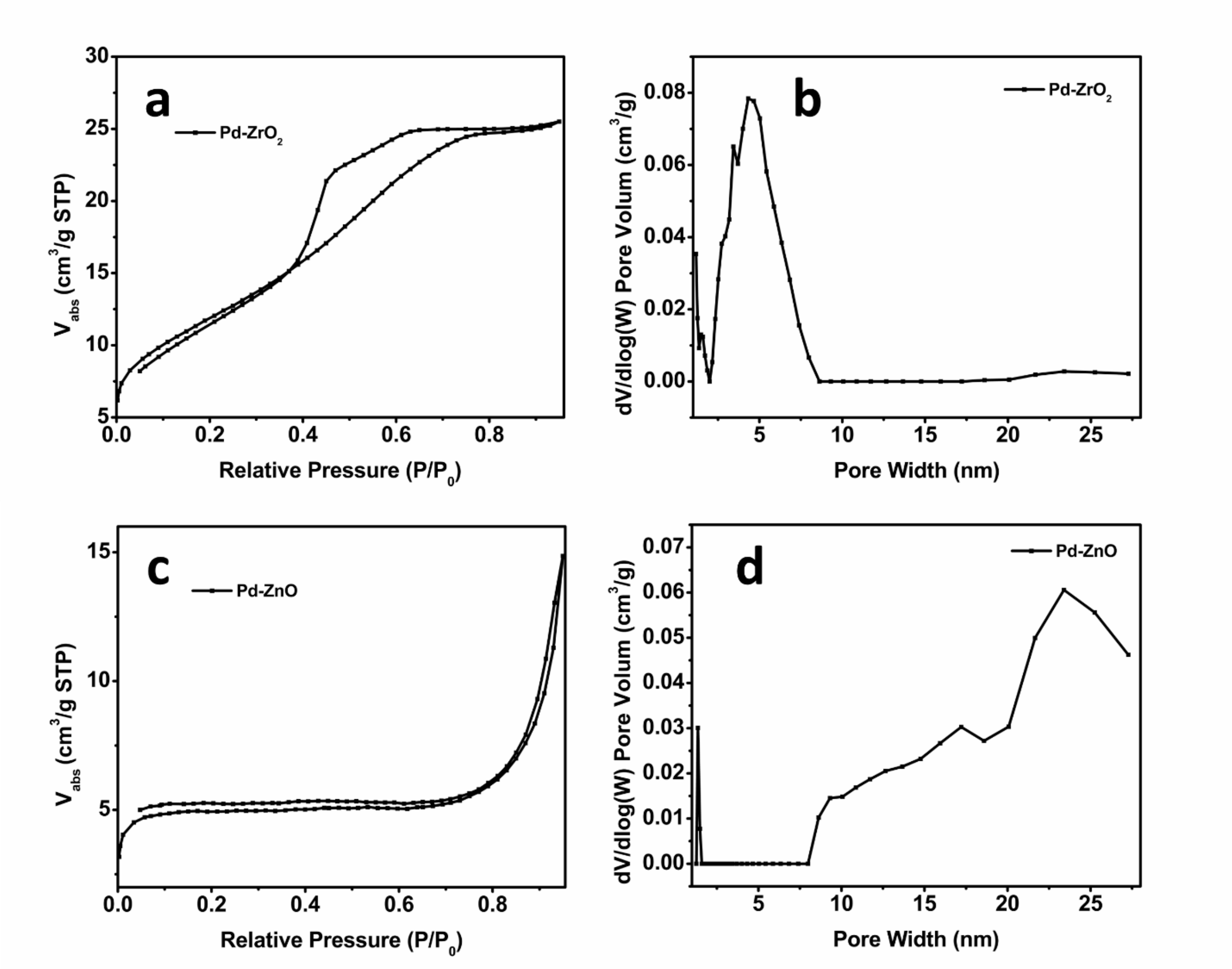

Supplementary Figure 3 N_2 adsorption-desorption isotherms and the pore size distributions of Pd-ZnO and Pd-ZrO_2. BET surface area is 18.15 m^2/g and 42.59 m^2/g for Pd-ZnO and Pd-ZrO_2, which is much lower then the Pd-ZnO-ZrO_2 (69.09 m^2/g)(Figure 1c). Pore volume is 0.023 cm^3/g and 0.039 cm^3/g for Pd-ZnO and Pd-ZrO_2.

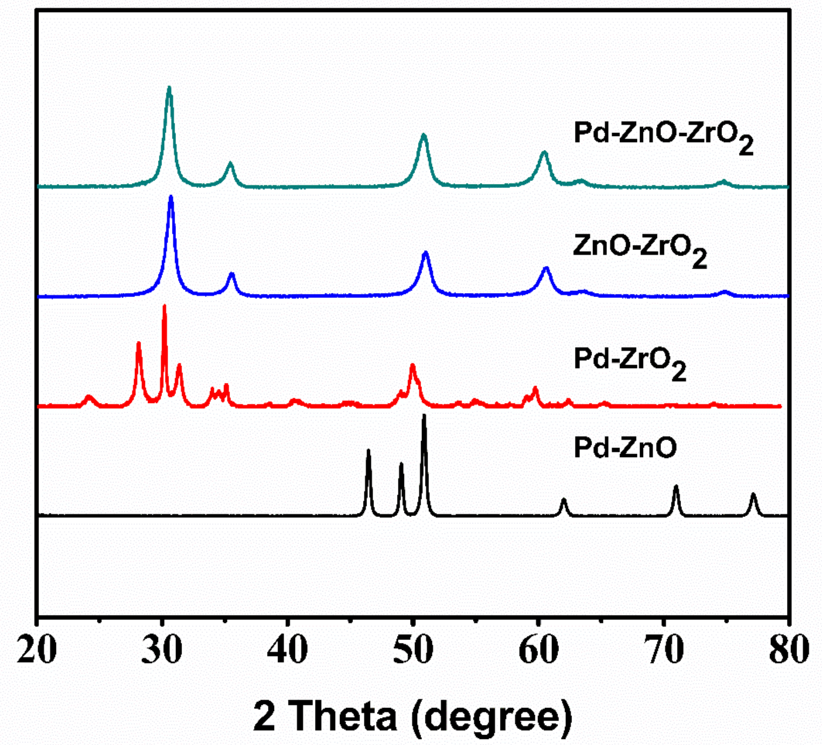

Supplementary Figure 4 XRD patterns of the Pd-ZnO-ZrO_2, ZnO-ZrO_2, Pd-ZrO_2 and Pd-ZnO. Pd-ZrO_2 is mainly in the monoclinic phase mixed with some in the tetragonal phase when ZnO was involved, a new phase formed attributed to the tetragonal ZrO_2 (011). Element distribution analysis shows that Zn is highly dispersed in ZrO_2 lattice matrix.[1]

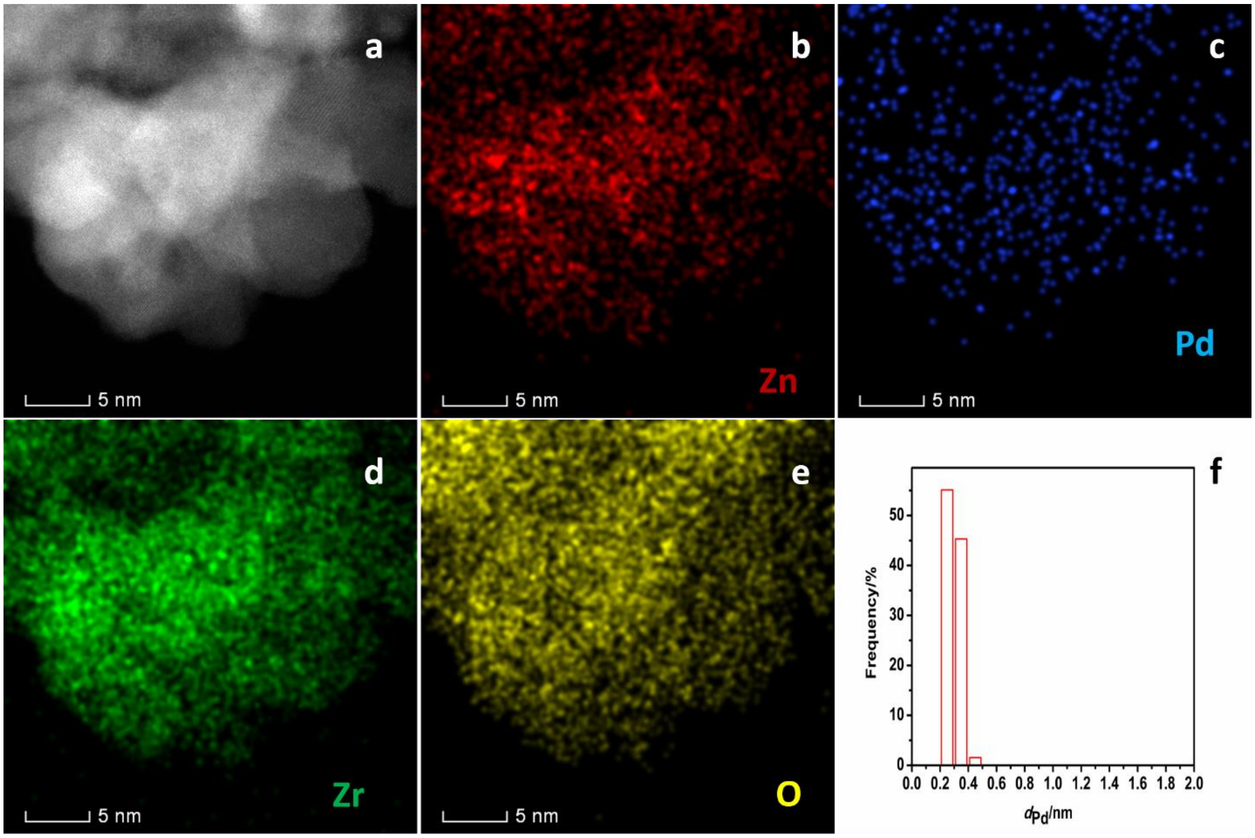

Supplementary Figure 5 (a-e) Images of HAADF-STEM and corresponding energy-dispersive X-ray spectroscopy (EDX) element mapping of the single atom catalyst Pd-ZnO-ZrO_2, Pd (blue), Zn (red), Zr (green) and O (yellow). (f) Pd size distribution (130 counts).

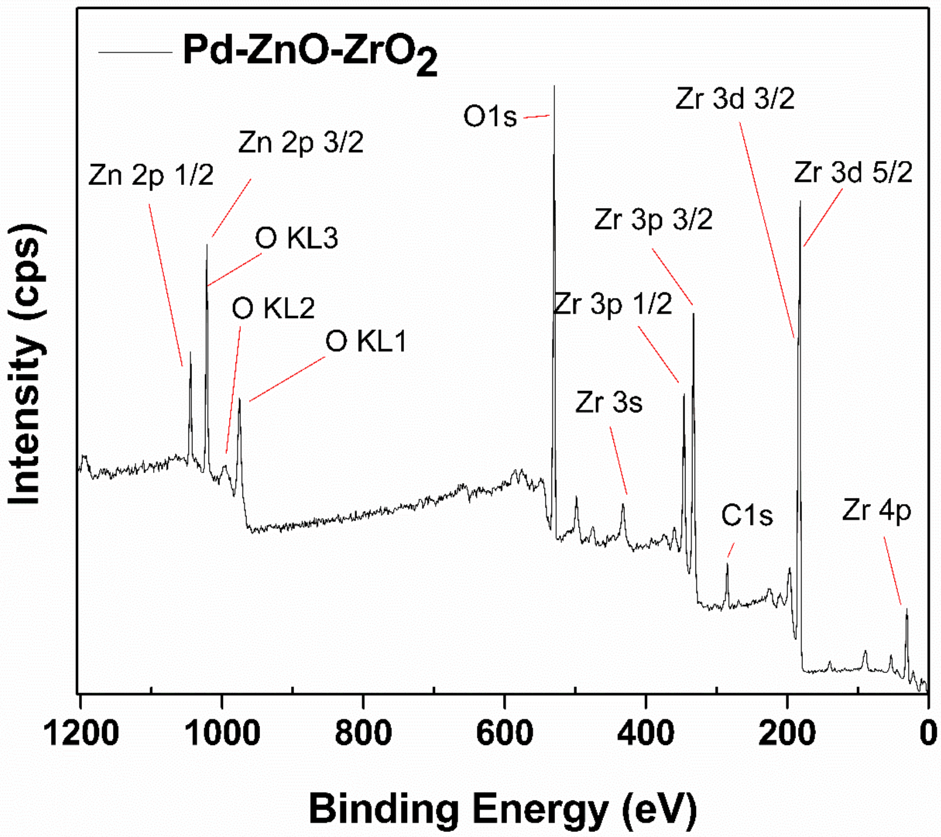

Supplementary Figure 6 XPS spectrum of the single atom catalyst Pd-ZnO-ZrO_2.

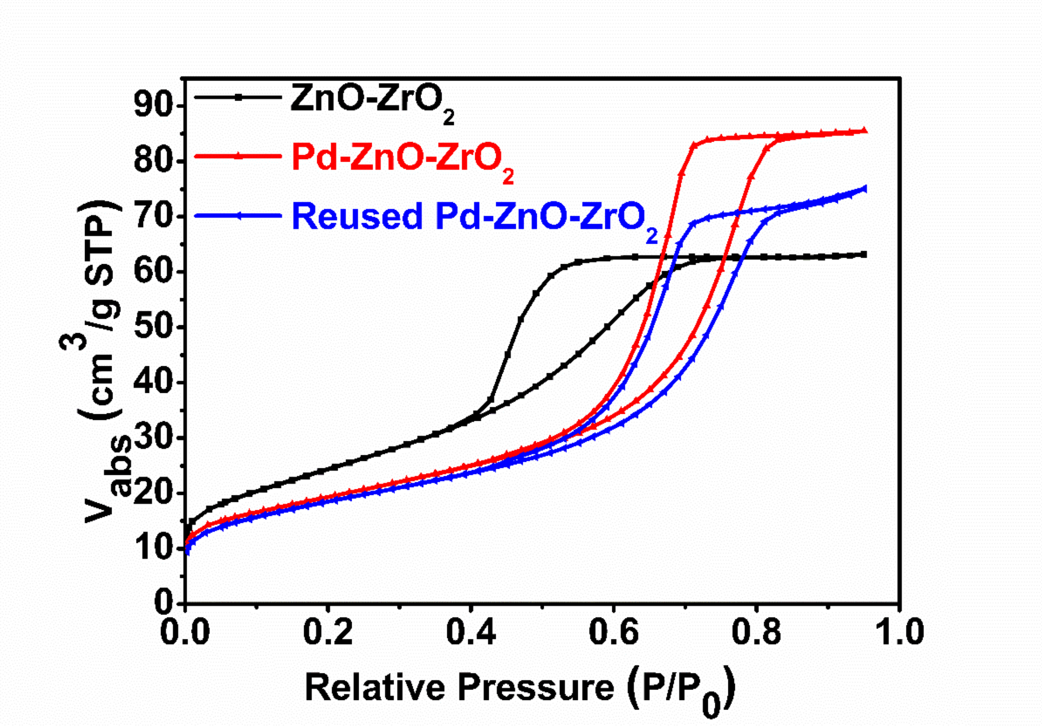

Supplementary Figure 7 Nitrogen isotherms of the fresh and reused catalyst, and the pristine support materials.

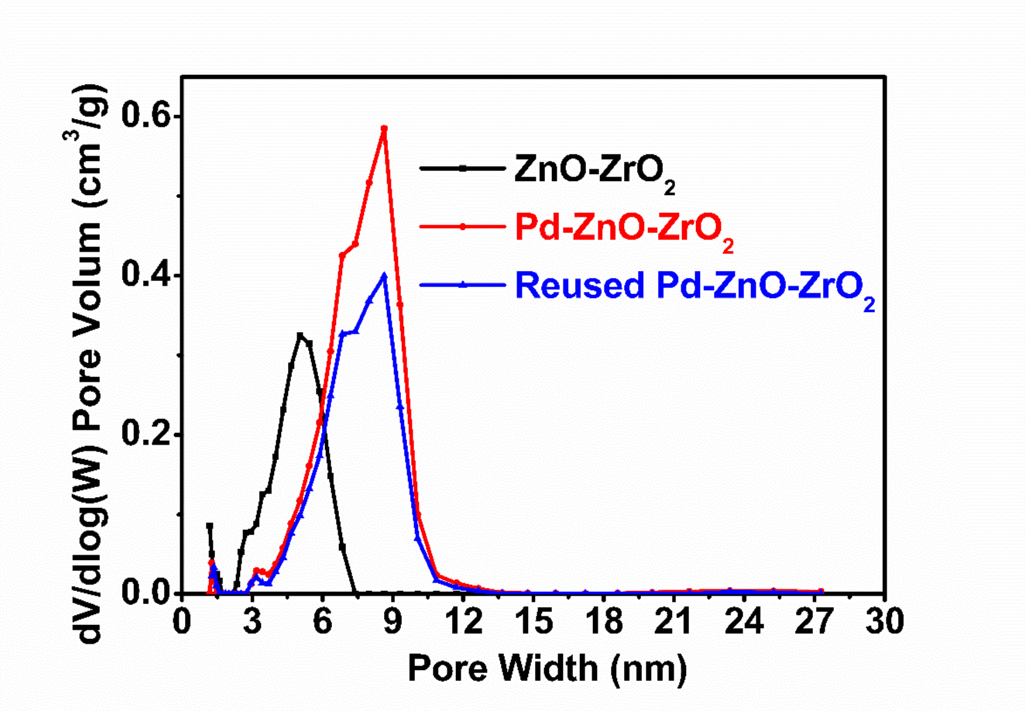

Supplementary Figure 8 Pore size distribution of the fresh and reused catalyst, and the pristine support materials.

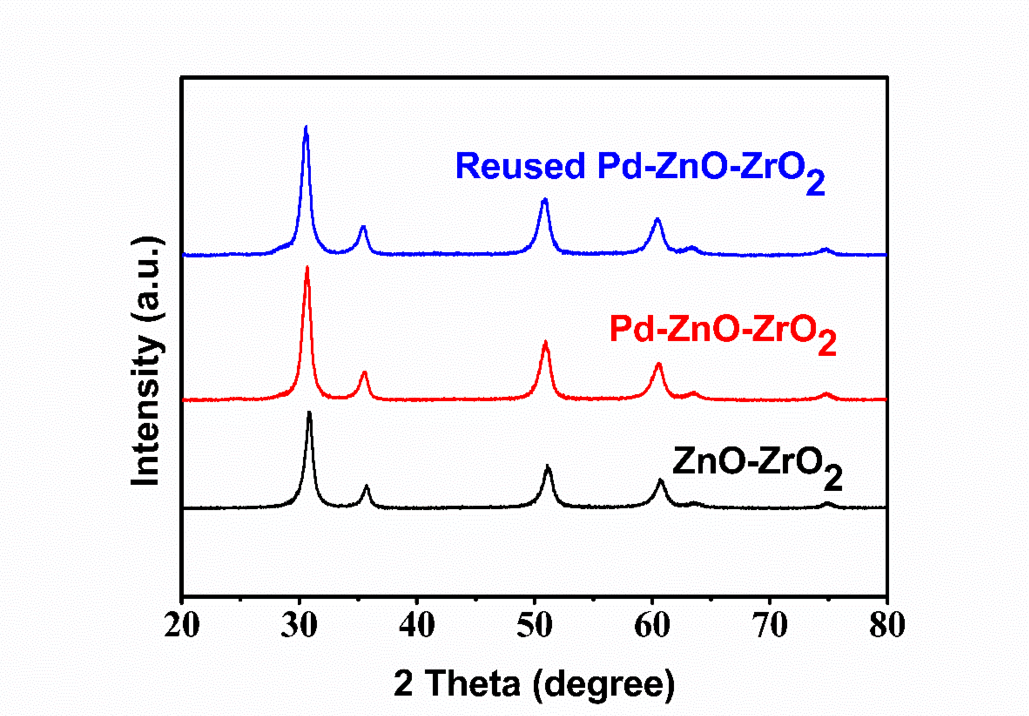

Supplementary Figure 9 XRD patterns of the fresh and reused catalyst, and the pristine support materials.

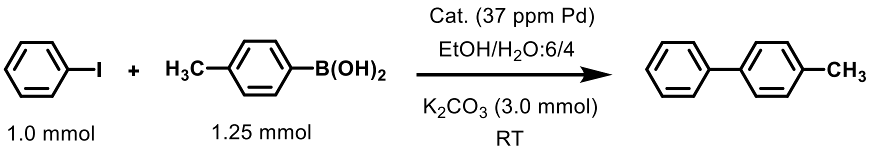

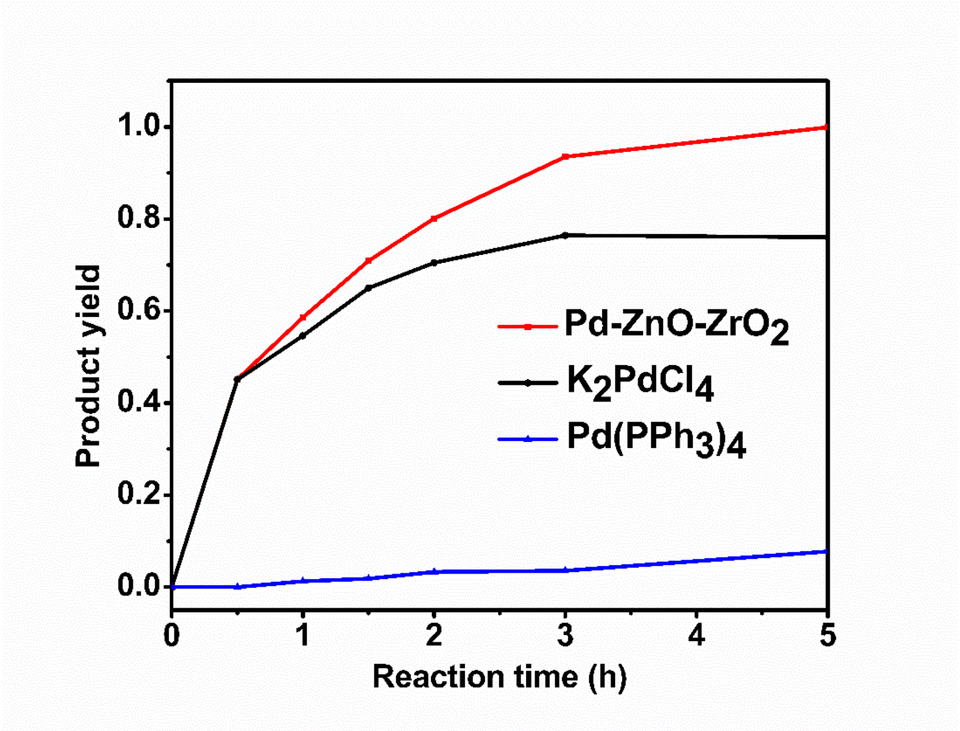

Supplementary Figure 10 Conversion-versus-time results of different Pd catalysts in Suzuki Coupling Reactions. To ensure the batch consistency, we did three parallel experiments of each reaction. The yield data are the average values of three independent experiments.

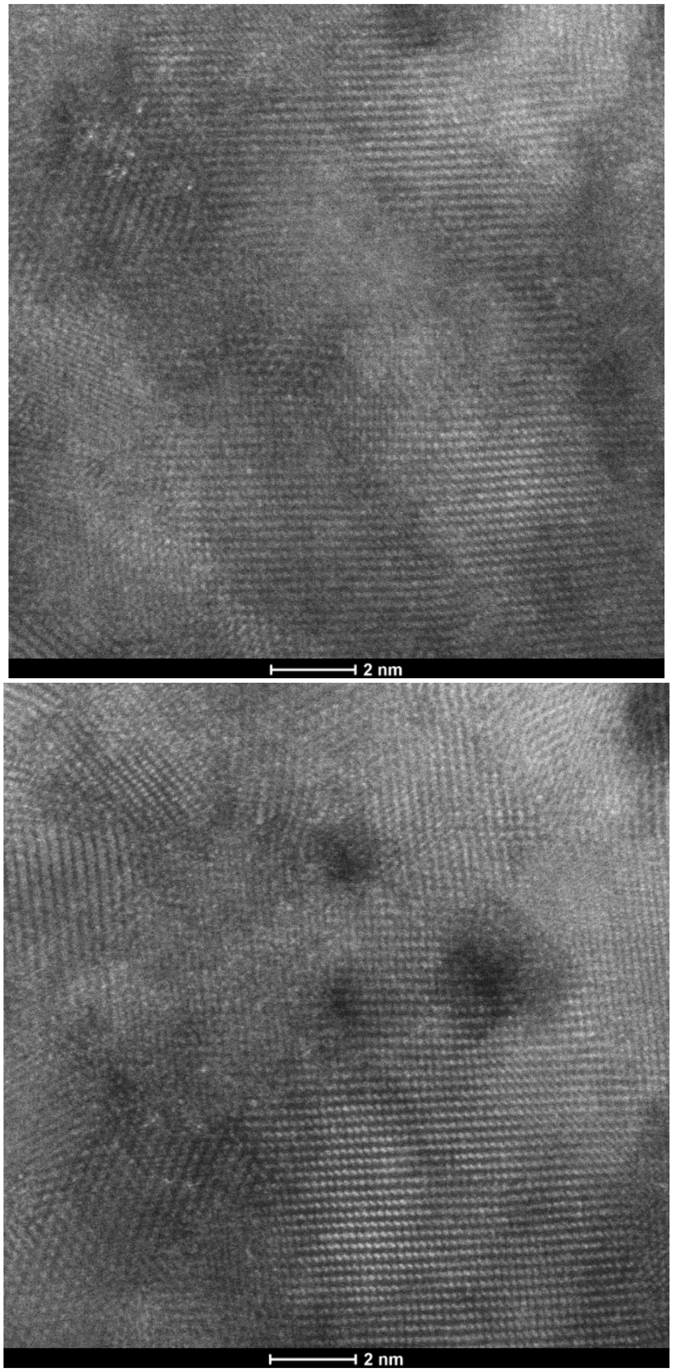

Supplementary Figure 11 Images of AC HAADF-STEM of the reused catalyst Pd-ZnO-ZrO_2. Atomically dispersed Pd atoms in the image can be clearly observed as light dots, which demonstrated the excellent stability of the catalyst.

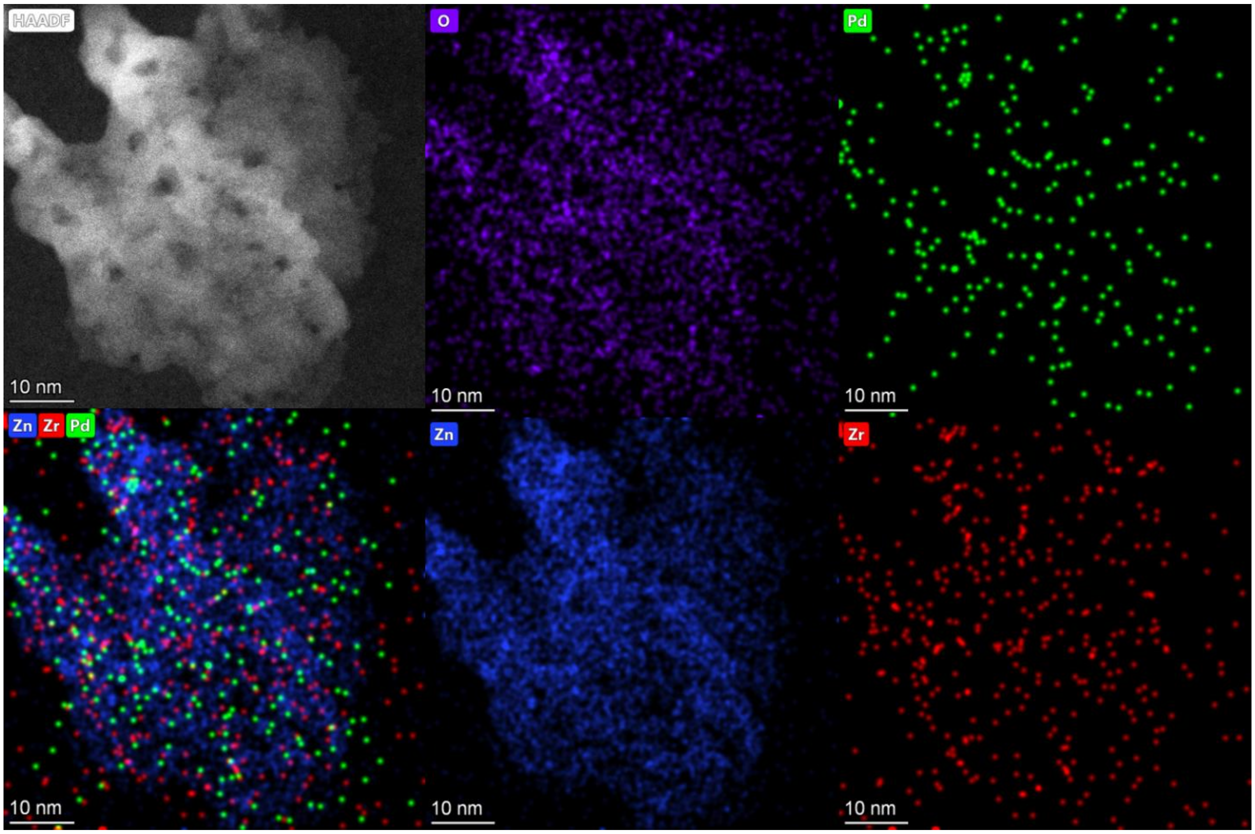

Supplementary Figure 12 Images of HAADF and corresponding EDX element mapping of the reused catalyst Pd-ZnO-ZrO_2. The results show that the Pd species existed evenly dispersed, neither sub-nanometer clusters nor nanoparticles were observed.

Supplementary Methods

3. Product characterization

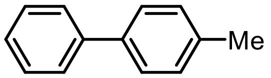

4-Methylbiphenyl (white solid, 181 mg, 93%)

^{1}H NMR (600 MHz, $CDCl_3$) δ 7.60-7.58 (m, 2 H), 7.52-7.50 (m, 2 H), 7.45-7.42 (m, 2 H), 7.35-7.32 (m, 1 H), 7.27-7.26 (m, 2 H), 2.41 (s, 3 H).
^{13}C NMR (150 MHz, $CDCl_3$) δ 141.32, 138.52, 137.16, 129.62, 128.85, 127.14, 127.13, 127.11, 21.24.

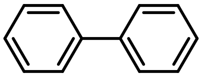

Biphenyl (white solid, 166 mg, 93%)

^{1}H NMR (600 MHz, $CDCl_3$) δ 7.63-7.61 (m, 2 H), 7.47-7.44 (m, 2 H), 7.38-7.35 (m, 1 H).
^{13}C NMR (150 MHz, $CDCl_3$) δ 141.40, 128.90, 128.89, 127.39, 127.31.

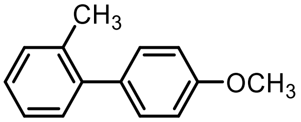

2-Methyl-4'-methoxy-1,1'-biphenyl (white solid, 172 mg, 87%)

^{1}H NMR (600 MHz, $CDCl_3$) δ 7.49-7.35 (m, 6 H), 7.09-7.06 (m, 2 H), 3.95 (s, 3 H), 2.41(s, 3 H).
^{13}C NMR (150 MHz, $CDCl_3$) δ 158.60, 158.31, 141.63, 135.52, 134.43, 130.39, 130.33, 129.99, 127.06, 125.86, 113.57, 55.28, 20.64.

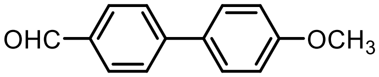

4-(4-Methoxyphenyl)benzaldehyde (white solid, 189 mg, 89%)

^{1}H NMR (600 MHz, $CDCl_3$) δ 10.04 (s, 1 H), 7.94-7.92 (d, J = 12.0 Hz, 2 H), 7.73-7.71 (d, J = 12.0 Hz, 2 H), 7.61-7.58 (m, 2 H), 7.02-7.00 (m, 2 H), 3.87 (s, 3 H).
^{13}C NMR (150 MHz, $CDCl_3$) δ 192.07, 160.25, 146.95, 134.81, 132.21, 130.48, 128.66, 127.21, 114.62, 55.55.

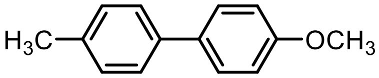

4-(4-Methylphenyl)-1-methoxybenzene (white solid, 184 mg, 93%)

^{1}H NMR (600 MHz, $CDCl_3$) 7.53-7.50 (m, 2 H), 7.46-7.44 (m, 2 H), 7.24-7.22 (m, 2 H), 6.98-6.96 (m, 2 H), 3.85 (s, 3 H), 2.39 (s, 3 H).
^{13}C NMR (150 MHz, $CDCl_3$) δ 159.05, 138.10, 136.50, 133.88, 129.58, 128.10, 126.72, 114.28, 55.48, 21.20.

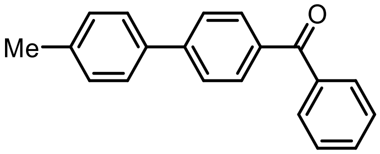

(4'-Methylbiphenyl-4-yl)(phenyl)methanone (white solid, 231 mg, 85%)

^{1}H NMR (600 MHz, $CDCl_3$) δ 7.83-7.76 (m, 4 H), 7.63-7.61 (m, 2 H), 7.54-7.42 (m, 5 H), 7.23-7.21(m, 2 H), 2.35 (s, 3 H).
^{13}C NMR (150 MHz, $CDCl_3$) δ 196.53, 145.34, 138.33, 137.97, 137.20, 136.08, 132.47, 130.90, 130.14, 129.85, 128.44, 127.28, 126.85, 21.33.

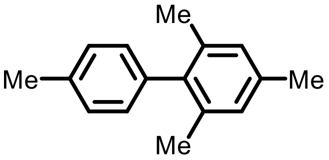

2,4,4',6-Tetramethylbiphenyl (white solid, 184 mg, 88%)

^{1}H NMR (600 MHz, $CDCl_3$) δ 7.33-7.32 (d, *J* = 6.0 Hz, 2 H), 7.14-7.13 (d, *J* = 6.0 Hz, 2 H), 7.05 (s, 2 H), 2.51 (s, 3 H), 2.44 (s, 3 H), 2.13 (s, 6 H).
^{13}C NMR (150 MHz, $CDCl_3$) δ 139.16, 128.17, 136.49, 136.23, 136.05, 129.28, 129.21, 128.16, 21.35, 21.14, 20.91.

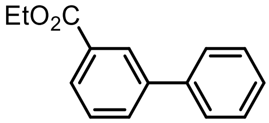

Ethyl 3-biphenylcarboxylate (white solid, 183 mg, 81%)

^{1}H NMR (600 MHz, $CDCl_3$) δ 8.31 (s, 1 H), 8.06-8.04 (m, 1 H), 7.80-7.78 (m, 1 H), 7.65 (m, 2 H), 7.53-7.46 (m, 3 H), 7.41-7.38 (m, 1 H), 4.45-4.41 (m, 2 H), 1.44-1.42 (m, 3 H).
^{13}C NMR (150 MHz, $CDCl_3$) δ 166.67, 141.55, 140.31, 131.54, 131.17, 128.99, 128.91, 128.43, 128.34, 127.82, 127.29, 127.27, 61.17, 14.48.

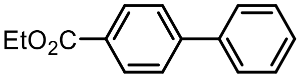

Ethyl 1,1'-biphenyl-4-carboxylate (white solid, 154 mg, 68%)

^{1}H NMR (600 MHz, $CDCl_3$) 8.13-8.11 (m, 2 H), 7.67-7.62 (m, 4 H), 7.49-7.46 (m, 2 H), 7.41-7.39 (t, 1 H), 4.43-4.40 (m, 2 H), 1.44-1.41 (t, 3 H).
^{13}C NMR (150 MHz, $CDCl_3$) δ 166.68, 145.67, 140.20, 130.20, 129.38, 129.06, 128.25, 127.43, 127.15, 61.13, 14.52.

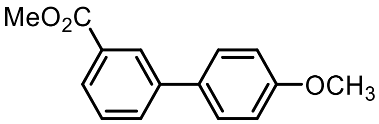

3-Biphenylcarboxylic acid, 4'-methoxy-, methyl ester (white solid, 198 mg, 82%)

^{1}H NMR (600 MHz, $CDCl_3$) 8.25-8.24 (m, 1 H), 7.98-7.96 (m, 1 H), 7.75-7.73 (m, 1 H), 7.57-7.55 (m, 2 H), 7.50-7.48 (m, 1 H), 7.01-6.99 (m, 2 H), 3.95 (s, 3 H), 3.86 (s, 3 H).
^{13}C NMR (150 MHz, $CDCl_3$) δ 167.27, 159.63, 141.18, 132.72, 131.19, 130.77, 128.93, 128.32, 127.91, 127.85, 114.46, 55.48, 52.28.

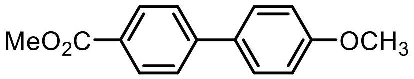

4-Biphenylcarboxylic acid, 4'-methoxy-, methyl ester (white solid, 183 mg, 76%)

^{1}H NMR (600 MHz, $CDCl_3$) δ 8.09-8.07 (d, *J* = 12.0 Hz, 2 H), 7.63-7.57 (m, 4 H), 7.01-6.99 (d, *J* = 12.0 Hz, 2 H), 3.94 (s, 3 H), 3.86 (s, 3 H).
^{13}C NMR (150 MHz, $CDCl_3$) δ 167.22, 160.00, 145.37, 132.57, 130.25, 128.51, 128.40, 126.62, 114.53, 55.53, 52.22.

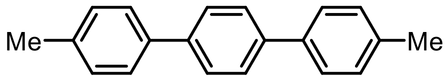

4,4''-Dimethyl-p-terphenyl (white solid, 222 mg, 86%)

^{1}H NMR (600 MHz, $CDCl_3$) δ 7.65 (s, 4 H), 7.55-7.53 (m, 4 H), 7.27-7.26 (m, 4 H), 2.41 (s, 6 H).
^{13}C NMR (150 MHz, $CDCl_3$) δ 139.93, 138.04, 137.22, 129.67, 127.40, 127.01, 21.27.

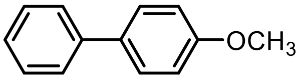

4-Methoxy-1,1'-biphenyl (white solid, 182 mg, 99%)

^{1}H NMR (600 MHz, $CDCl_3$) δ 7.57-7.53 (m, 4 H), 7.43-7.41 (m, 2 H), 7.32-7.31 (m, 1 H), 7.00-6.98 (m, 2 H), 3.86 (s, 3 H).
^{13}C NMR (150 MHz, $CDCl_3$) δ 159.30, 140.98, 133.94, 128.86, 128.30, 126.88, 126.80, 114.35, 55.49.

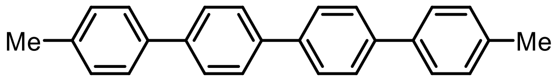

4,4'''-Dimethyl-1,1':4',1'':4'',1'''-quaterphenyl (white solid, 178 mg, 69%)

^{1}H NMR (600 MHz, $CDCl_3$) δ 7.71-7.63 (m, 6 H), 7.57-7.51 (m, 6 H), 7.29-7.26 (m, 4 H), 2.41 (s, 6 H).
^{13}C NMR (150 MHz, $CDCl_3$) δ 139.93, 138.04, 137.22, 132.06, 129.73, 129.70, 129.68, 128.74, 127.57, 127.48, 127.47, 127.40, 127.02, 127.01, 21.27.

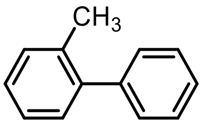

2-Methylbiphenyl (white solid, 156 mg, 93%)

^{1}H NMR (600 MHz, $CDCl_3$) δ 7.49-7.45 (m, 2 H), 7.42-7.38 (m, 2 H), 7.34-7.30 (m, 4 H), 2.34 (s, 3 H).
^{13}C NMR (150 MHz, $CDCl_3$) δ 142.09, 142.06, 135.46, 130.43, 129.92, 129.32, 128.19, 127.37, 126.88, 125.89, 20.61.

4. Copies of ^{1}H NMR and ^{13}C NMR Spectra

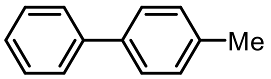

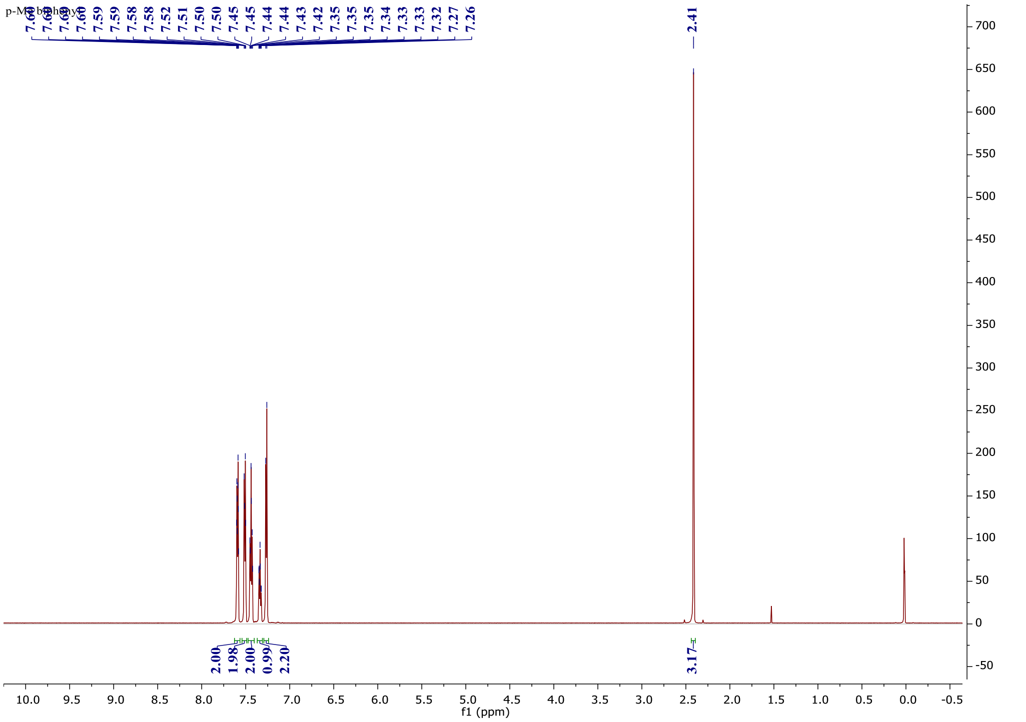

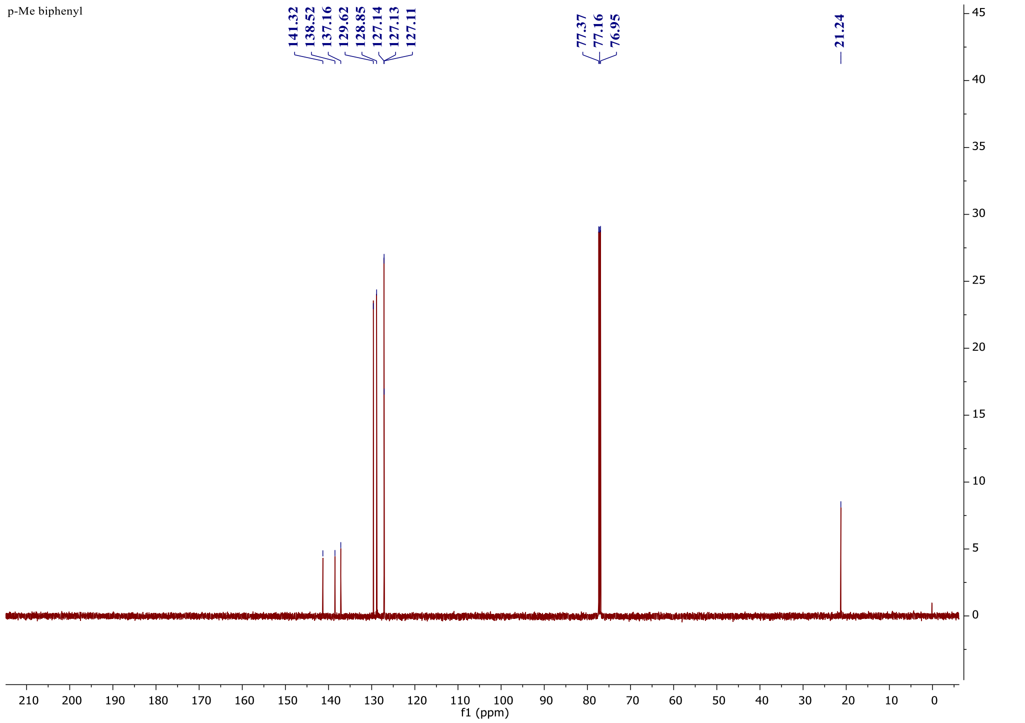

Supplementary Figure 13. ^{1}H and ^{13}C NMR spectra of 4-Methylbiphenyl

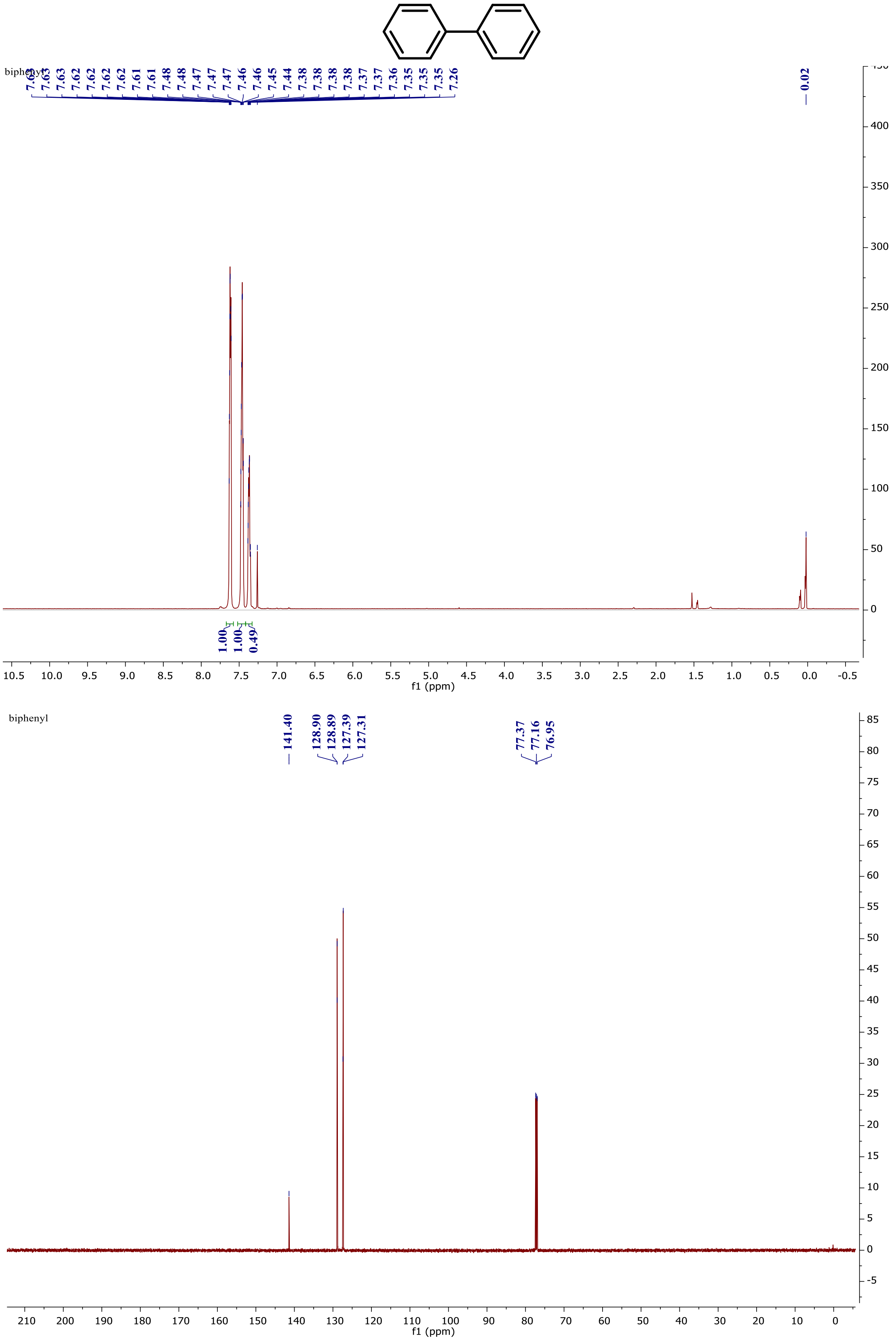

Supplementary Figure 14. ^{1}H and ^{13}C NMR spectra of biphenyl

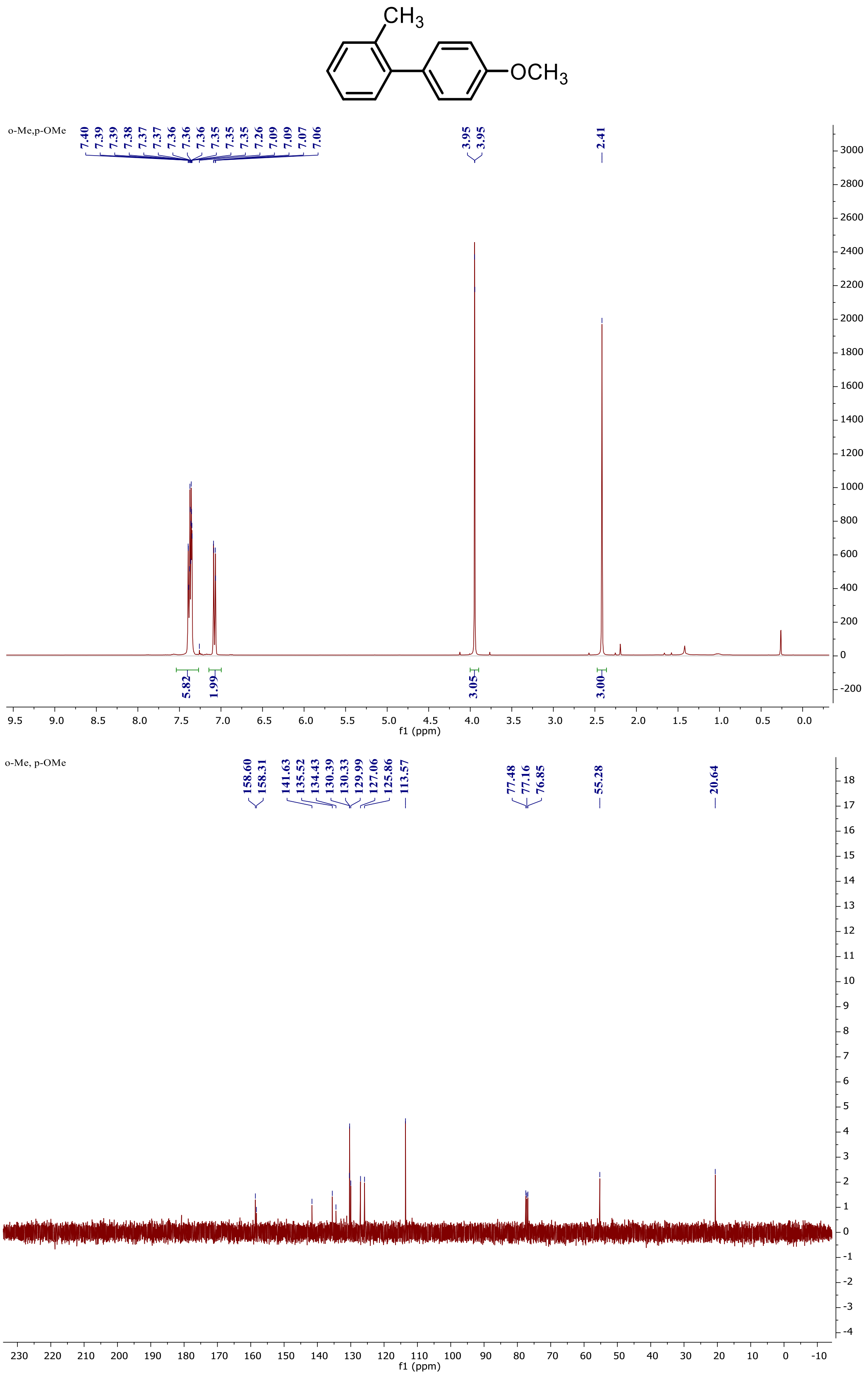

Supplementary Figure 15. ^{1}H and ^{13}C NMR spectra of 2-Methyl-4'-methoxy-1,1'-biphenyl

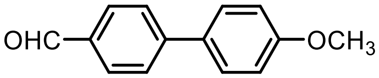

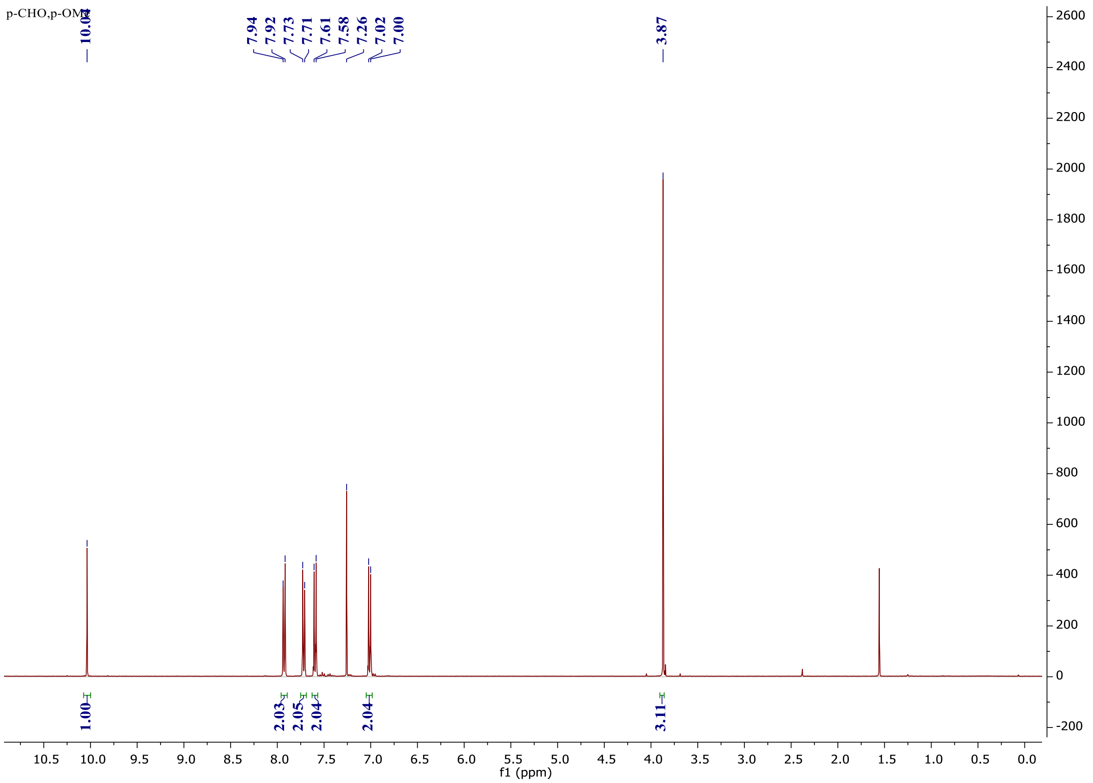

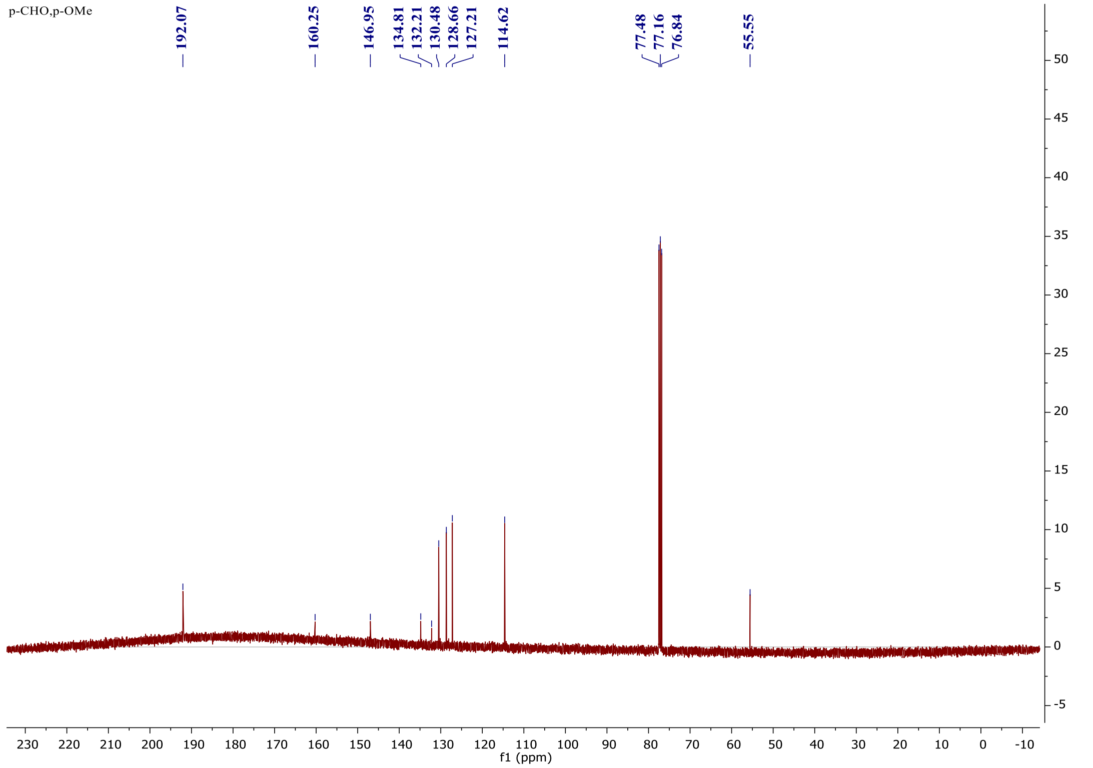

Supplementary Figure 16. ^{1}H and ^{13}C NMR spectra of 4-(4-Methoxyphenyl)benzaldehyde

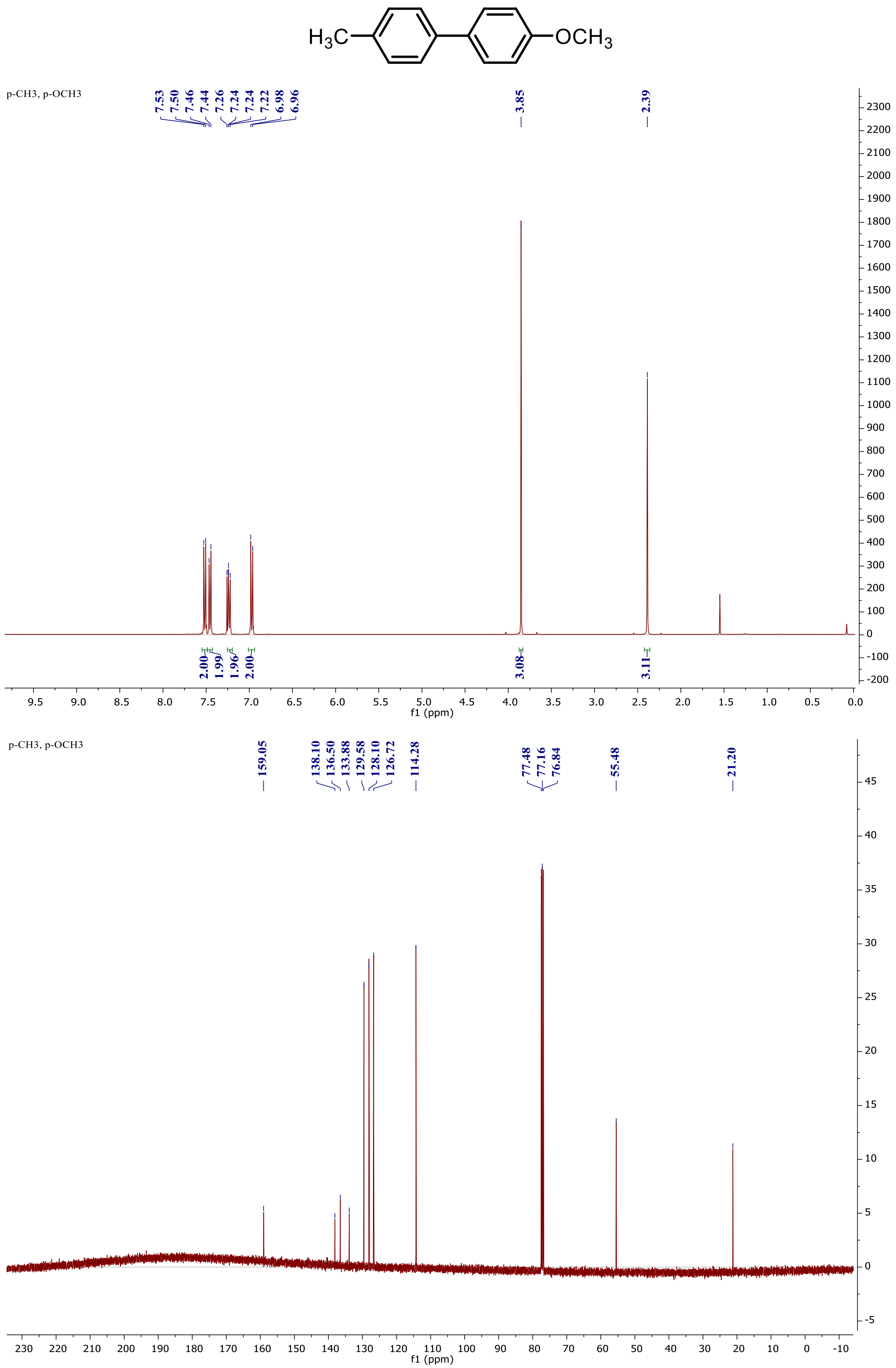

Supplementary Figure 17. ^{1}H and ^{13}C NMR spectra of 4-(4-Methylphenyl)-1-methoxybenzene

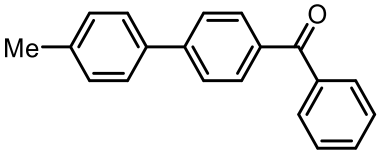

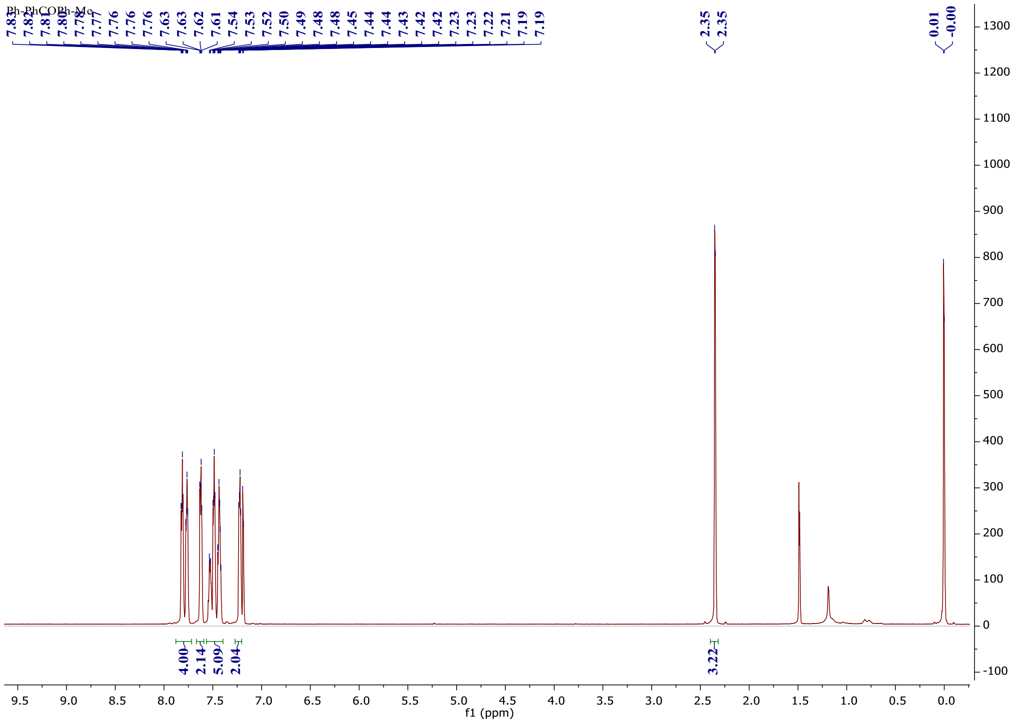

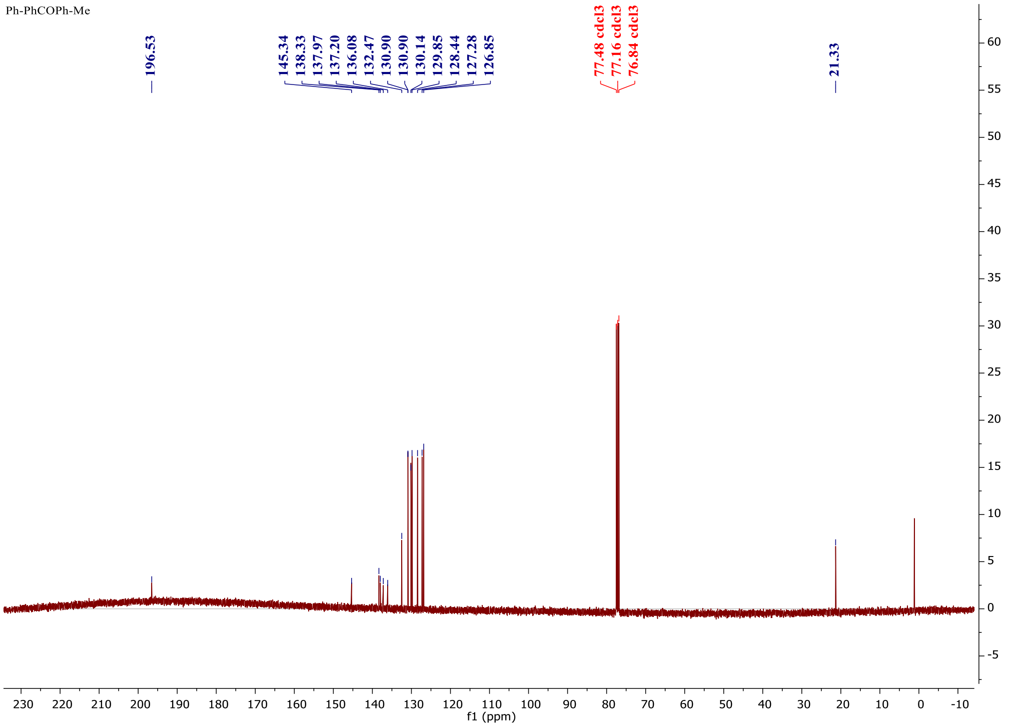

Supplementary Figure 18. ^{1}H and ^{13}C NMR spectra of (4'-Methylbiphenyl-4-yl)(phenyl)methanone

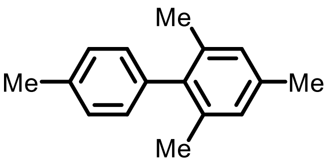

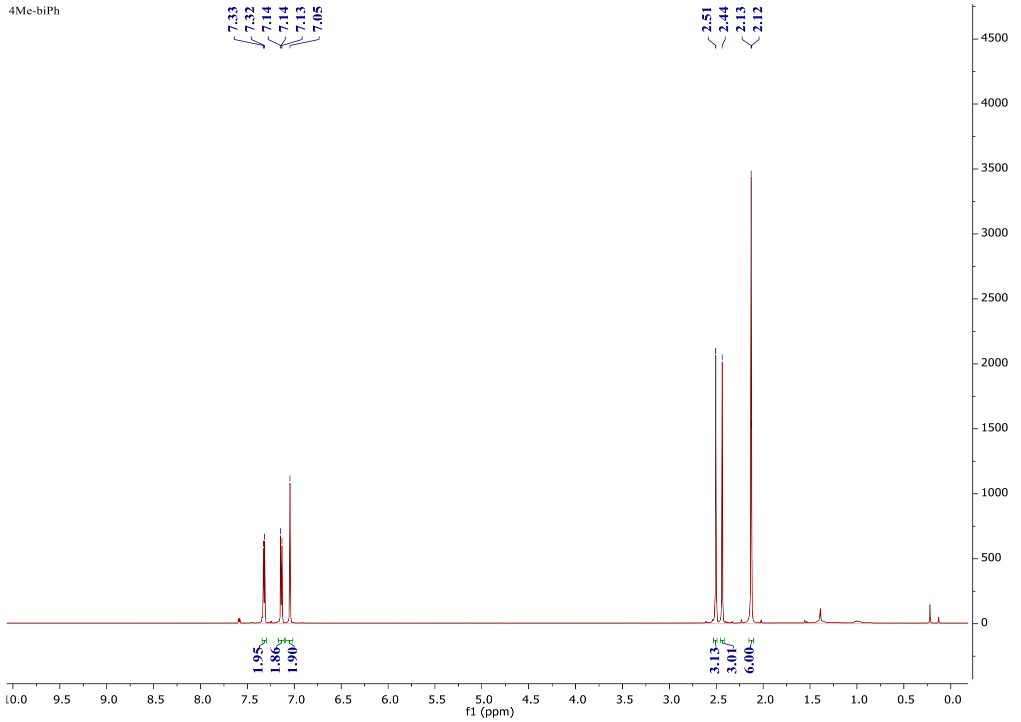

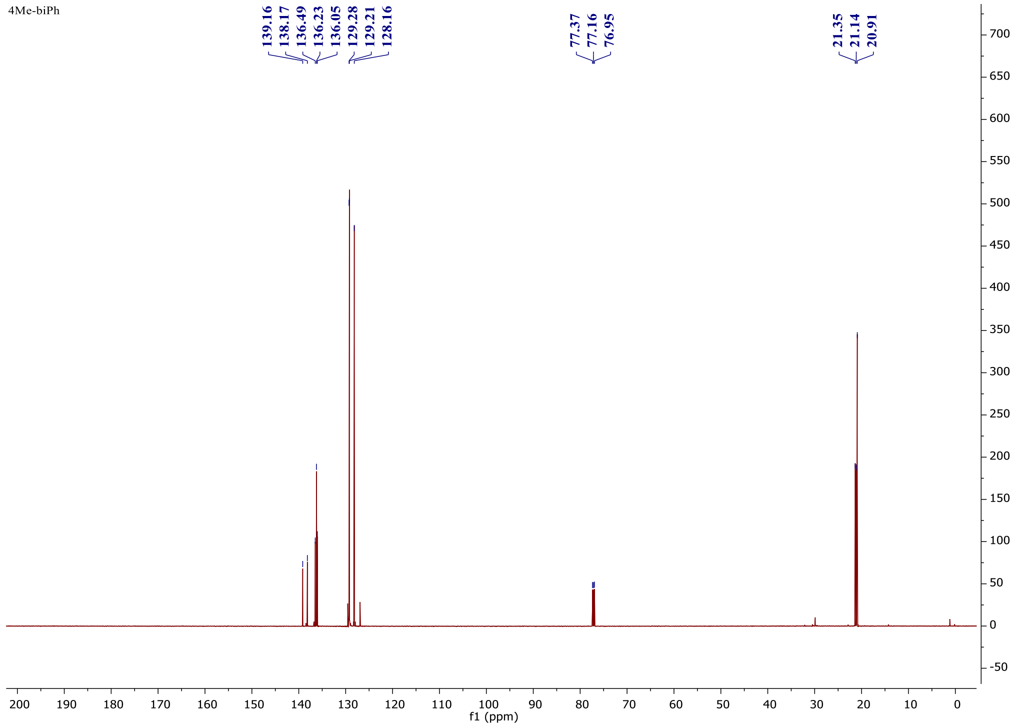

Supplementary Figure 19. ^{1}H and ^{13}C NMR spectra of 2,4,4',6-Tetramethylbiphenyl

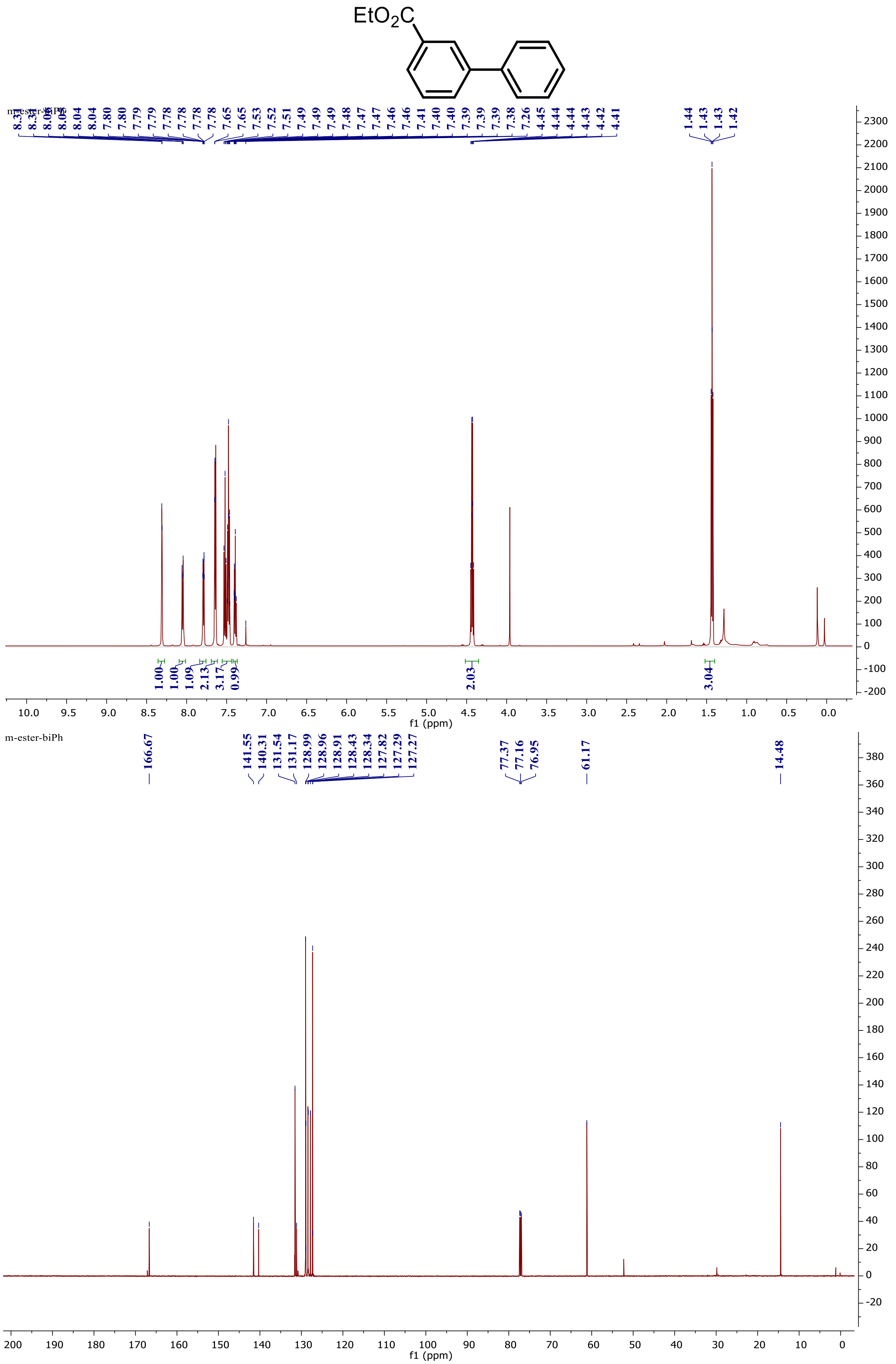

Supplementary Figure 20. ^{1}H and ^{13}C NMR spectra of Ethyl 3-biphenylcarboxylate

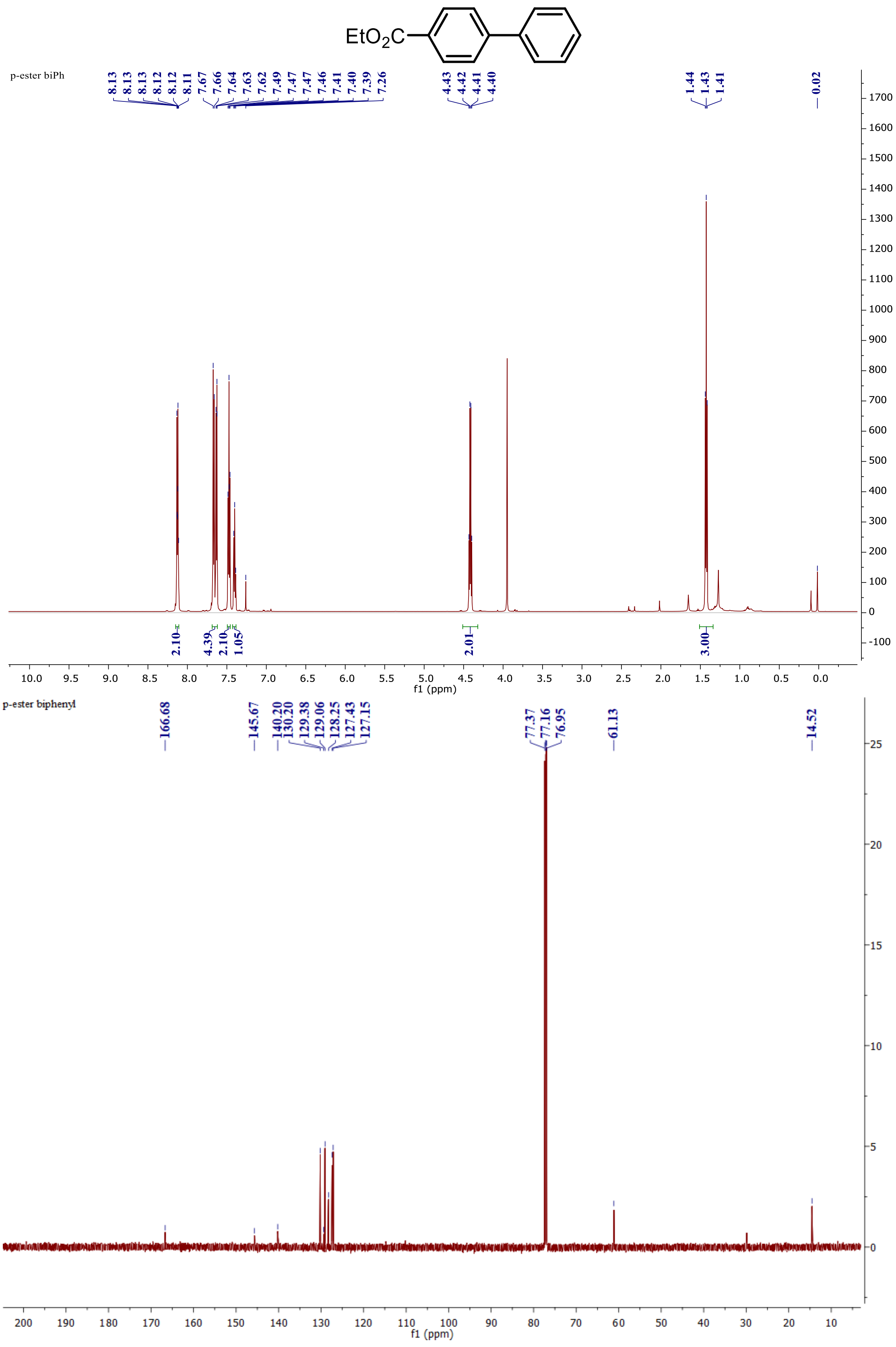

Supplementary Figure 21. ^{1}H and ^{13}C NMR spectra of Ethyl 1,1'-biphenyl-4-carboxylate

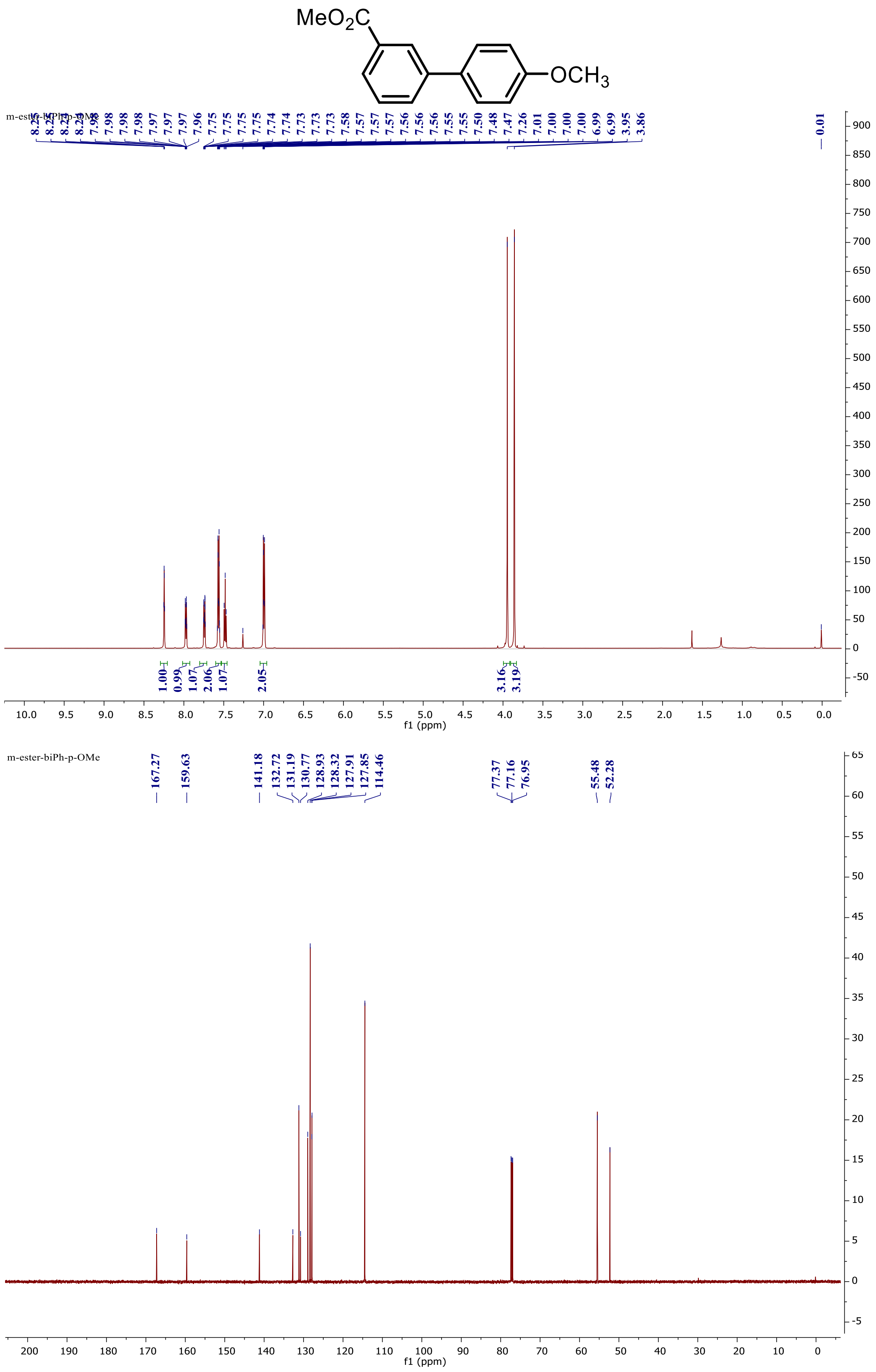

Supplementary Figure 22. ^{1}H and ^{13}C NMR spectra of (3-Biphenylcarboxylic acid, 4'-methoxy-, methyl ester)

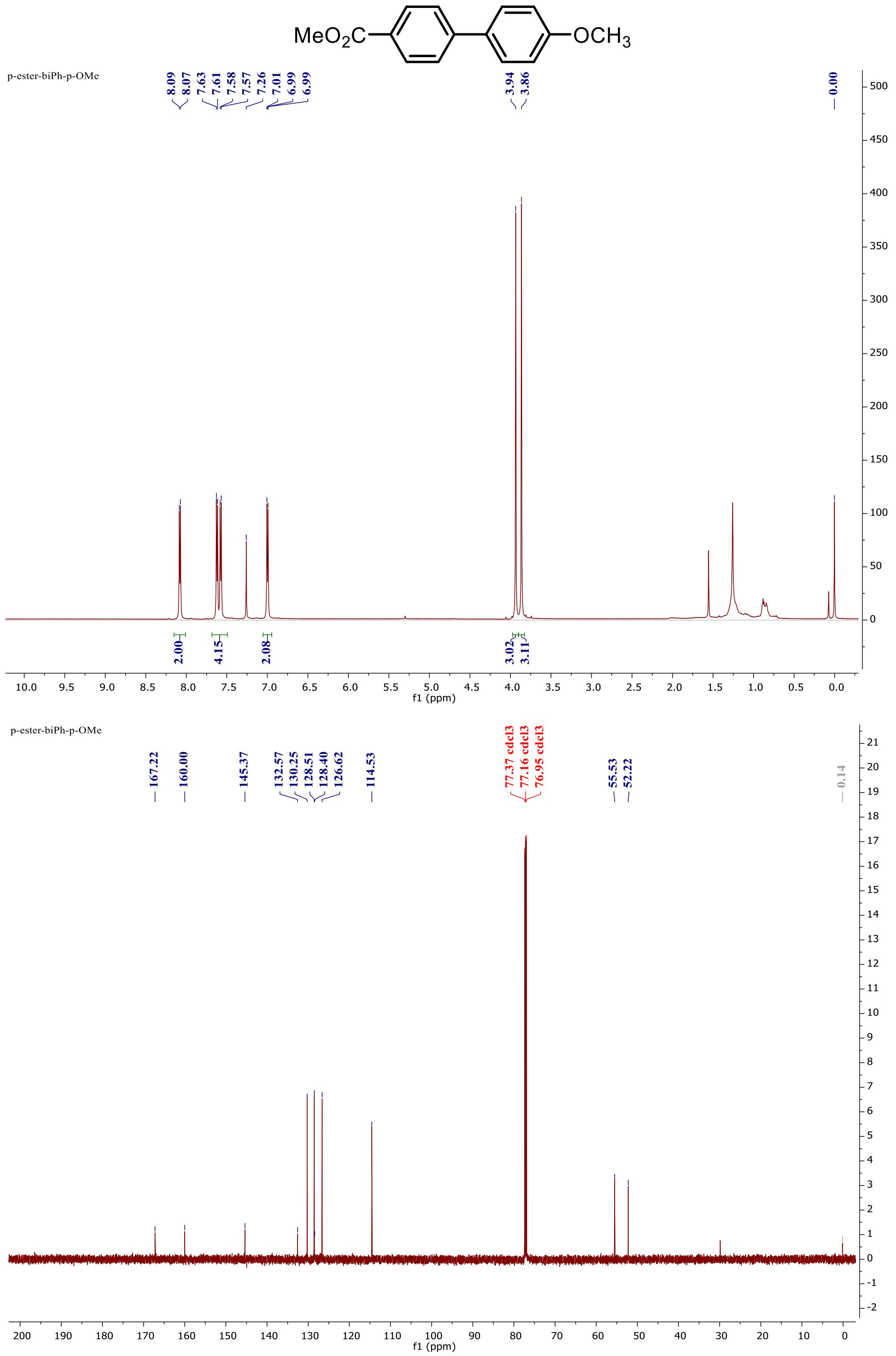

Supplementary Figure 23. ^{1}H and ^{13}C NMR spectra of 4-Biphenylcarboxylic acid, 4'-methoxy-, methyl ester

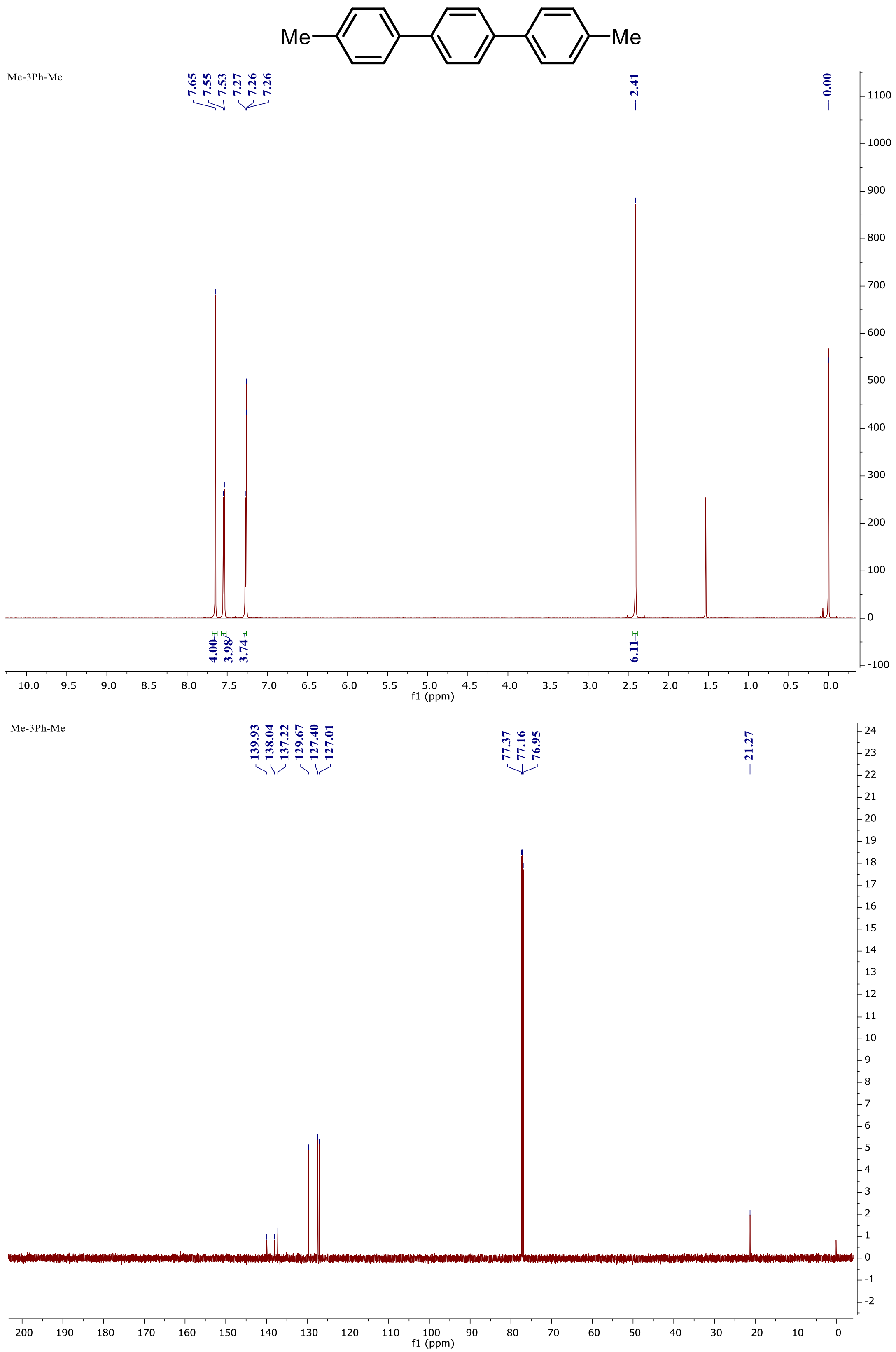

Supplementary Figure 24. ^{1}H and ^{13}C NMR spectra of 4,4''-Dimethyl-p-terphenyl

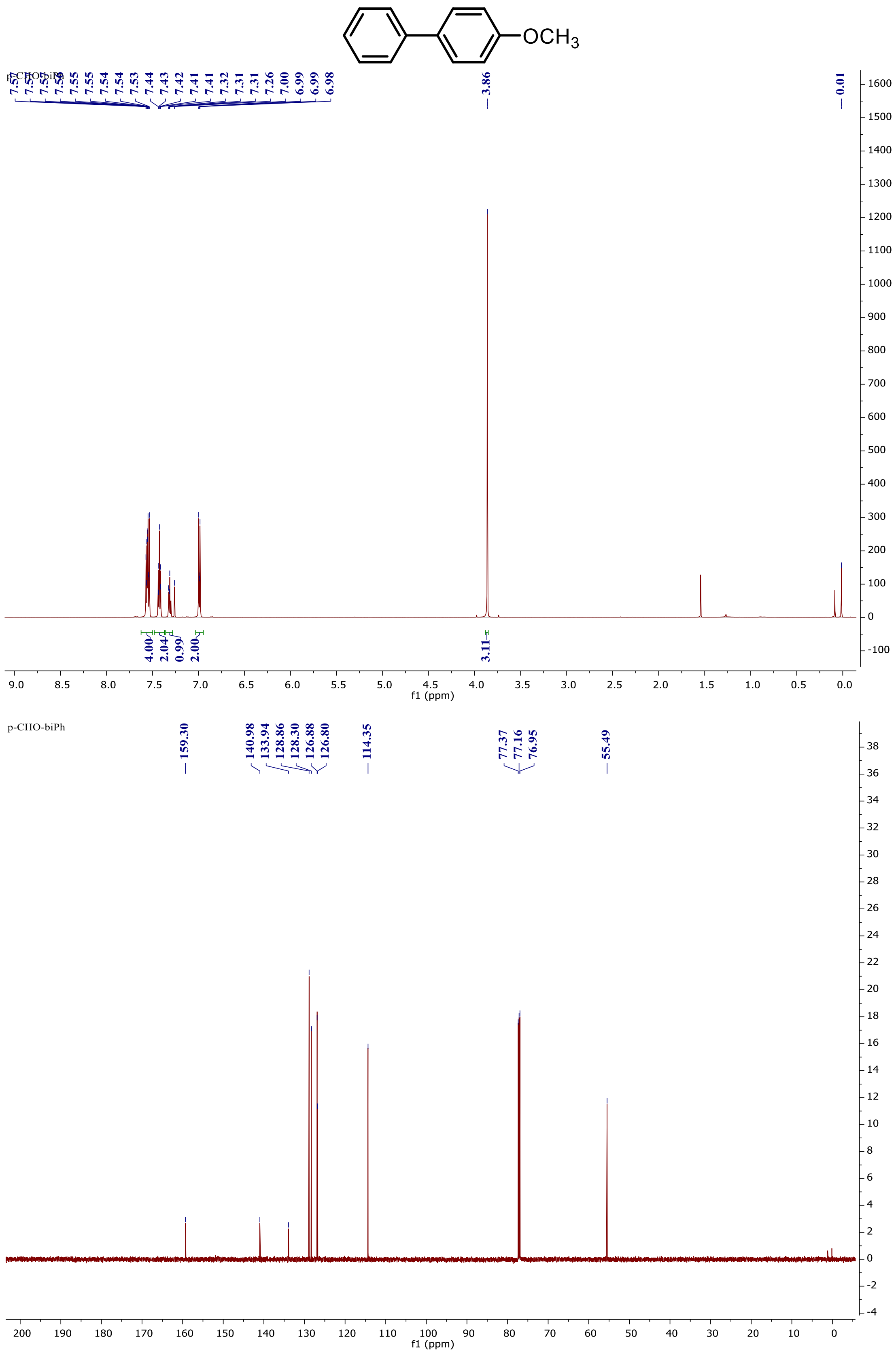

Supplementary Figure 25. ^{1}H and ^{13}C NMR spectra of 4-Methoxy-1,1'-biphenyl

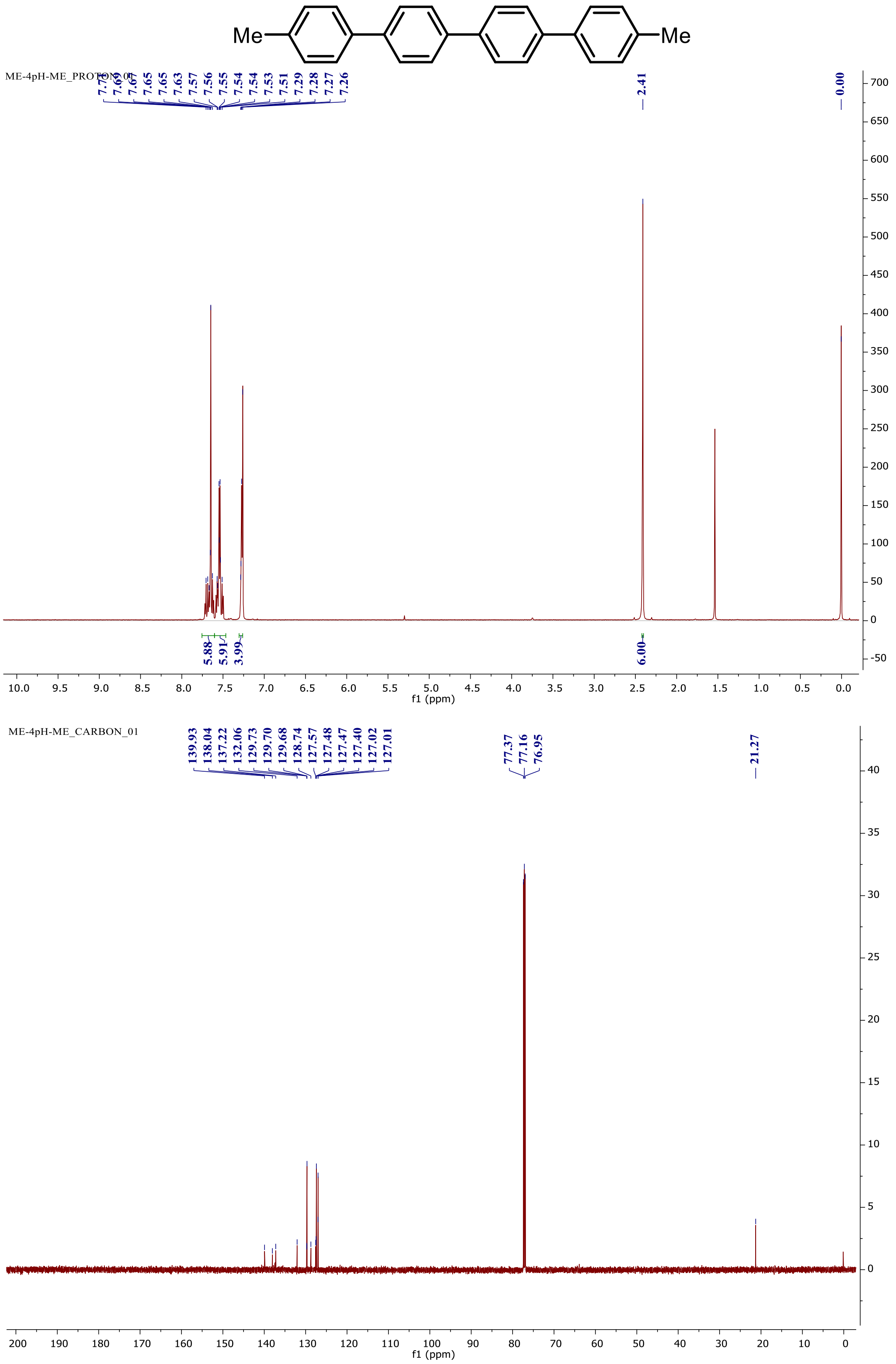

Supplementary Figure 26. ^{1}H and ^{13}C NMR spectra of 4,4'''-Dimethyl-1,1':4',1'':4'',1'''-quaterphenyl

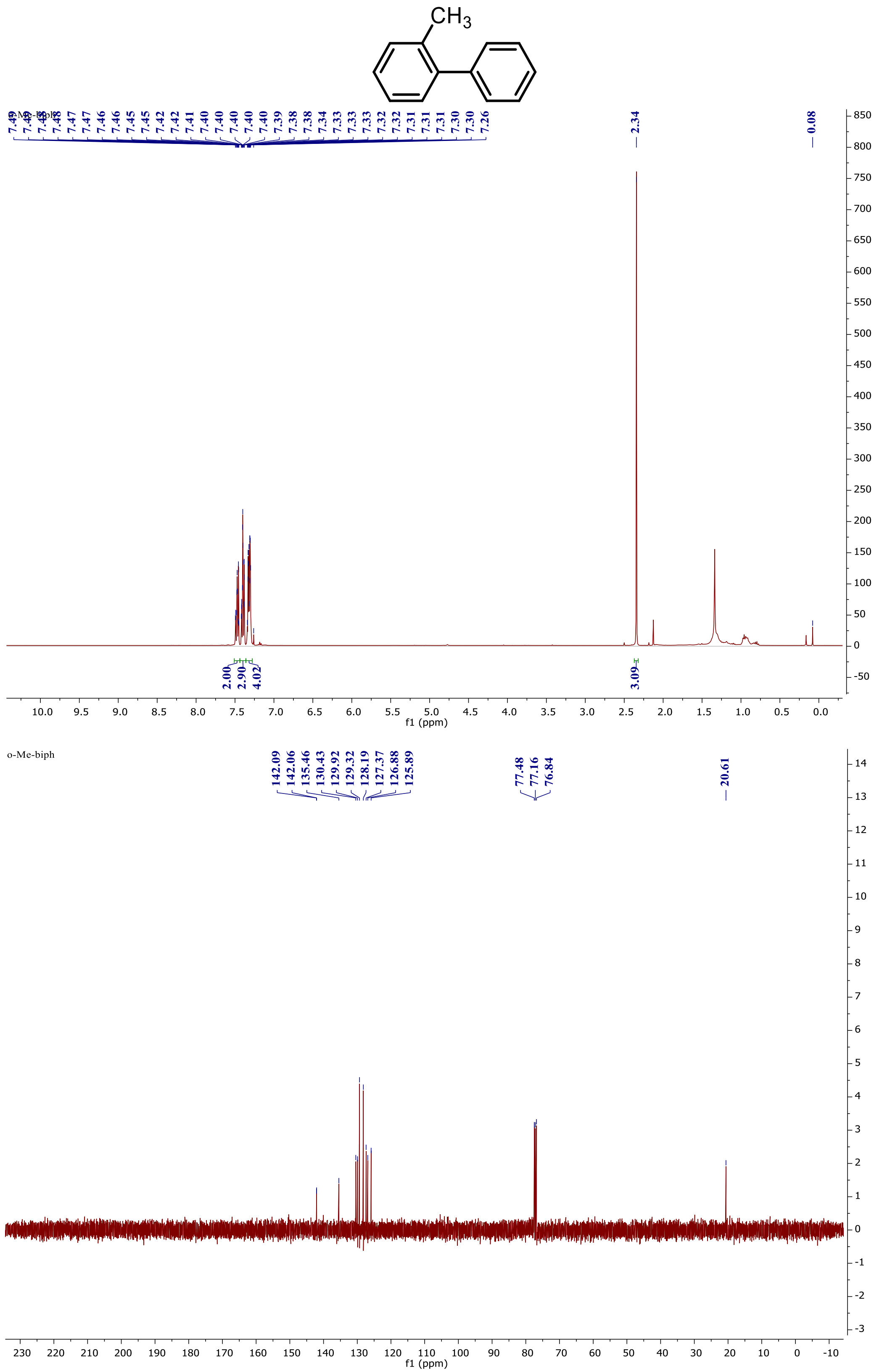

Supplementary Figure 27. ^{1}H and ^{13}C NMR spectra of 2-Methylbiphenyl
